# Single Nucleotide Polymorphism Microarray Analysis Unveils Copy‐Number Abnormalities and Genetic Heterogeneity in Malaysian Childhood B‐Cell Precursor Acute Lymphoblastic Leukemia

**DOI:** 10.1002/mgg3.70182

**Published:** 2026-03-02

**Authors:** Nor Soleha Mohd Dali, Nursaedah Abdullah Aziz, Muhamad Farid Zulkifle, Durar Aqilah Zamri, Nor Rizan Kamaluddin, Seoh‐Leng Yeoh, Betty Lee‐Sue Ho, Nazzlin Dizana Din, Azly Sumanty Ab Ghani, Wan Amal Hayati Wan Hassan, Zubaidah Zakaria, Ezalia Esa, Yuslina Mat Yusoff

**Affiliations:** ^1^ Cancer Research Centre Institute for Medical Research, National Institutes of Health, Ministry of Health Selangor Malaysia; ^2^ Genetics Department Hospital Tunku Azizah, Ministry of Health Kuala Lumpur Malaysia; ^3^ Department of Pediatrics Penang General Hospital, Ministry of Health Penang Malaysia; ^4^ Department of Pediatrics Hospital Umum Sarawak, Ministry of Health Sarawak Malaysia; ^5^ Department of Pediatrics Hospital Sultanah Nur Zahirah, Ministry of Health Terengganu Malaysia; ^6^ Department of Pathology Hospital Sultanah Nur Zahirah, Ministry of Health Terengganu Malaysia; ^7^ Cytogenetics & Molecular Diagnostics Laboratory Pantai Premier Pathology Kuala Lumpur Malaysia

**Keywords:** B‐cell precursor acute lymphoblastic leukemia, copy‐number abnormalities, MLPA, pediatric, SNP 6.0 microarray

## Abstract

**Introduction:**

B‐cell precursor acute lymphoblastic leukemia (BCP‐ALL) is a prevalent pediatric hematologic malignancy characterized by diverse chromosomal aberrations that significantly influence its prognosis. This study aimed to comprehensively characterize the genomic landscape of BCP‐ALL in 55 Malaysian patients with BCP‐ALL.

**Methods:**

Single‐nucleotide polymorphism (SNP) 6.0 microarray and multiplex ligation‐dependent probe amplification were utilized to characterize and validate copy‐number abnormalities involving key oncogenes, respectively.

**Results:**

The SNP 6.0 microarray identified 191 copy‐number abnormalities in 55 patients, including common subtypes such as hyperdiploidy (*n* = 14/191, 7.3%), hypodiploidy (*n* = 2/191, 1.1%), and various copy‐number abnormalities such as interstitial (23.0%), terminal (17.2%), focal (37.1%), and intragenic (18.0%). Notably, intrachromosomal amplification of chromosome 21 (iAMP21) was not observed, suggesting its rarity in this cohort. Comparison with conventional cytogenetic techniques, including Trypsin‐Leishman's banding karyotyping, fluorescent in situ hybridization (FISH), and reverse transcription‐polymerase chain reaction (RT‐PCR), revealed superior resolution of the SNP 6.0 microarray in detecting submicroscopic copy‐number abnormalities. Furthermore, MLPA confirmed abnormalities in several oncogenes, including *CDKN2A/B*, *EBF1*, *ERG*, *ETV6*, *IKZF1*, *JAK2*, and *PAX5*.

**Conclusion:**

This study demonstrates the utility of combined SNP 6.0 microarray and MLPA in providing a comprehensive and refined understanding of the genetic landscape of BCP‐ALL in the Malaysian population. This understanding may facilitate risk stratification and the development of personalized treatment strategies.

## Graphical abstract

B‐cell precursor acute lymphoblastic leukemia (BCP‐ALL) is a prevalent pediatric hematologic malignancy characterized by diverse chromosomal aberrations that significantly influence its prognosis. This study aimed to comprehensively characterize the genomic landscape of childhood BCP‐ALL in a cohort of 55 Malaysian patients.

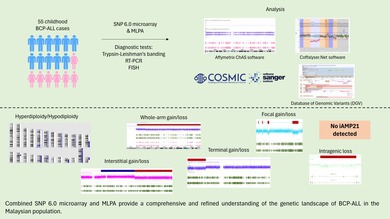

## 1 Introduction

B‐cell precursor acute lymphoblastic leukemia (BCP‐ALL) is a blood malignancy that commonly occurs among children and adolescents and accounts for a significant proportion of childhood cancer mortality (Pagliaro et al. 2024). This subtype of ALL makes up approximately 80% of ALL cases worldwide (Pagliaro et al. 2024). BCP‐ALL knowingly burdens the childhood population in Malaysia, being the seventh most common cancer in the Malaysian population. This demands a comprehensive understanding of its genetic landscape (National Cancer Registry Department, National Cancer Institute 2024). Acquired chromosomal abnormalities are the hallmark of BCP‐ALL, defining biologically distinct subtypes of the disease. Although significant advancements in treatment regimens have led to improved survival rates, the underlying genetic heterogeneity of BCP‐ALL plays an essential role in risk stratification, prognosis, and treatment outcome (Lejman et al. 2022).

Risk stratification is crucial in BCP‐ALL management, as it guides treatment decisions and may influence therapeutic outcomes. Several diagnostic tests are available for risk stratification and prognosis of BCP‐ALL, including Trypsin‐Leishman's banding karyotyping and fluorescence in situ hybridization (FISH). However, these tests have limitations, such as the inability to detect submicroscopic chromosomal abnormalities, the need for high‐quality bone marrow aspirates, and being time‐consuming, which require highly skilled personnel for analysis (Zhou et al. 2021). In addition, reverse transcription‐polymerase chain reaction (RT‐PCR) is also used to detect BCP‐ALL translocation. However, the limitations of RT‐PCR include the inability to detect abnormal transcripts that are not covered by the design panel, high susceptibility to RNA degradation, and the risk of false‐positive results due to sample cross‐contamination (Dolz et al. 2013; Jennings et al. 2014).

Recurring chromosomal copy‐number abnormalities (CNAs), particularly those affecting key signaling pathways and impacting therapeutic outcomes, are critical for risk stratification in BCP‐ALL. Some of the key CNAs in BCP‐ALL risk stratification include hyperdiploidy, hypodiploidy, copy‐number deletions involving BCP‐ALL‐related genes such as *IKZF1*, *PAX5*, *JAK2*, *KMT2A*, and intrachromosomal amplification of chromosome 21 (iAMP21) (Arber et al. 2016; Duffield et al. 2023; Lejman et al. 2022). Single‐nucleotide polymorphism (SNP) 6.0 microarray and multiplex ligation‐dependent probe amplification (MLPA) techniques have been documented to enhance the detection of these abnormalities, improving the precision of risk assessment and guiding tailored therapeutic strategies (Bashton et al. 2020). The SNP 6.0 microarray provides high‐resolution genome‐wide analysis, allowing the detection of copy‐number variations (CNVs), loss of heterozygosity (LOH), and regions of homozygosity (Camuset et al. 2023; Creasey et al. 2021; Gourmel et al. 2024). On the other hand, MLPA is a targeted technique used to quantify the copy‐number of specific genes, providing valuable information on the status of key oncogenes and tumor suppressor genes. This study used the SNP 6.0 microarray and MLPA to comprehensively describe the genetic landscape, particularly CNAs, in Malaysian patients with childhood BCP‐ALL. By identifying the spectrum of CNAs, we will better understand the genetic landscape and heterogeneity of BCP‐ALL in the Malaysian childhood population and inform the local clinical settings for risk stratification and personalized treatment strategies.

## 2 Materials and Methods

### 2.1 Ethical Compliance

This study was approved by the Malaysian Medical Research and Ethics Committee (MREC) (NMRR‐16‐1468‐32133). Informed consent was obtained from the patients.

### 2.2 Study Samples

This study used archived diagnostic bone marrow aspirate (BMA) samples sent to the Institute for Medical Research (IMR) for cytogenetic and molecular tests, including Trypsin‐Leishman's banding karyotyping, FISH, and RT‐PCR for prognosis and risk stratification. A total of 55 samples from patients with newly diagnosed BCP‐ALL between January 1, 2016, and December 31, 2017, were included. The patients were treated according to UK Pediatric ALL (UKALL) 2003 (Vora et al. 2010) or ALL‐Berlin‐Frankfurt‐Münster (BFM) 95, 2002, or 2009 (Castillo et al. 2009) Protocols. Some patients (*n* = 21/55, 38.2%) were also followed up for their treatment outcomes from diagnosis until December 2019 (Table S1). Only samples at diagnosis underwent SNP 6.0 microarray and MLPA analyses, as our laboratory did not receive treated samples.

### 2.3 Cytogenetics, FISH, and RT‐PCR Analysis

Bone marrow aspirate samples were cytogenetically analysed using Trypsin‐Leishman's banding karyotyping at diagnosis (Data S1). FISH was performed on good‐quality samples using probes *ETV6/RUNX1* ES dual‐color translocation probe (Vysis‐Abbott Molecular Inc., Illinois, USA), *KMT2A/MLL* Dual‐Color Break Apart Rearrangement (Vysis‐Abbott Molecular), and *CRLF2* Break‐Apart probe (Cytocell Ltd., Cambridge, UK) (Data S1). The *ETV6/RUNX1* translocation was identified as the most common translocation in childhood BCP‐ALL. Due to the known variability of *MLL* rearrangements, RT‐PCR analysis was complemented using *KMT2A/MLL* break‐apart probe. Concurrently, the *CRLF2* break‐apart probe was employed for the detection of hyperdiploidy. RT‐PCR (HemaVision‐28 N, DNA Diagnostics, Denmark) was performed according to the manufacturer's instructions to identify specific gene rearrangements. Among the 28 chromosome translocations detected by this kit include t(1;19)(q23;p13) (*TCF3‐PBX1*), t(9,22)(q34;q11) (*BCR‐ABL1*), t(12;21)(p13;q22) (*ETV6‐RUNX1*), t(16;21)(p11;q22) (*FUS‐ERG*) (the full list of the chromosome translocations is listed in Data S1).

### 2.4 Genomic DNA Extraction

Genomic DNA was extracted from the BMA using the QIAamp Blood Midi Kit (Qiagen, Germantown, MD) before being subjected to SNP 6.0 microarray and MLPA analysis. The genomic DNA was quantified using a NanoDrop spectrophotometer (Thermo Fisher Scientific, MA, USA).

### 2.5 SNP 6.0 Microarray Analysis

**FIGURE 1:**
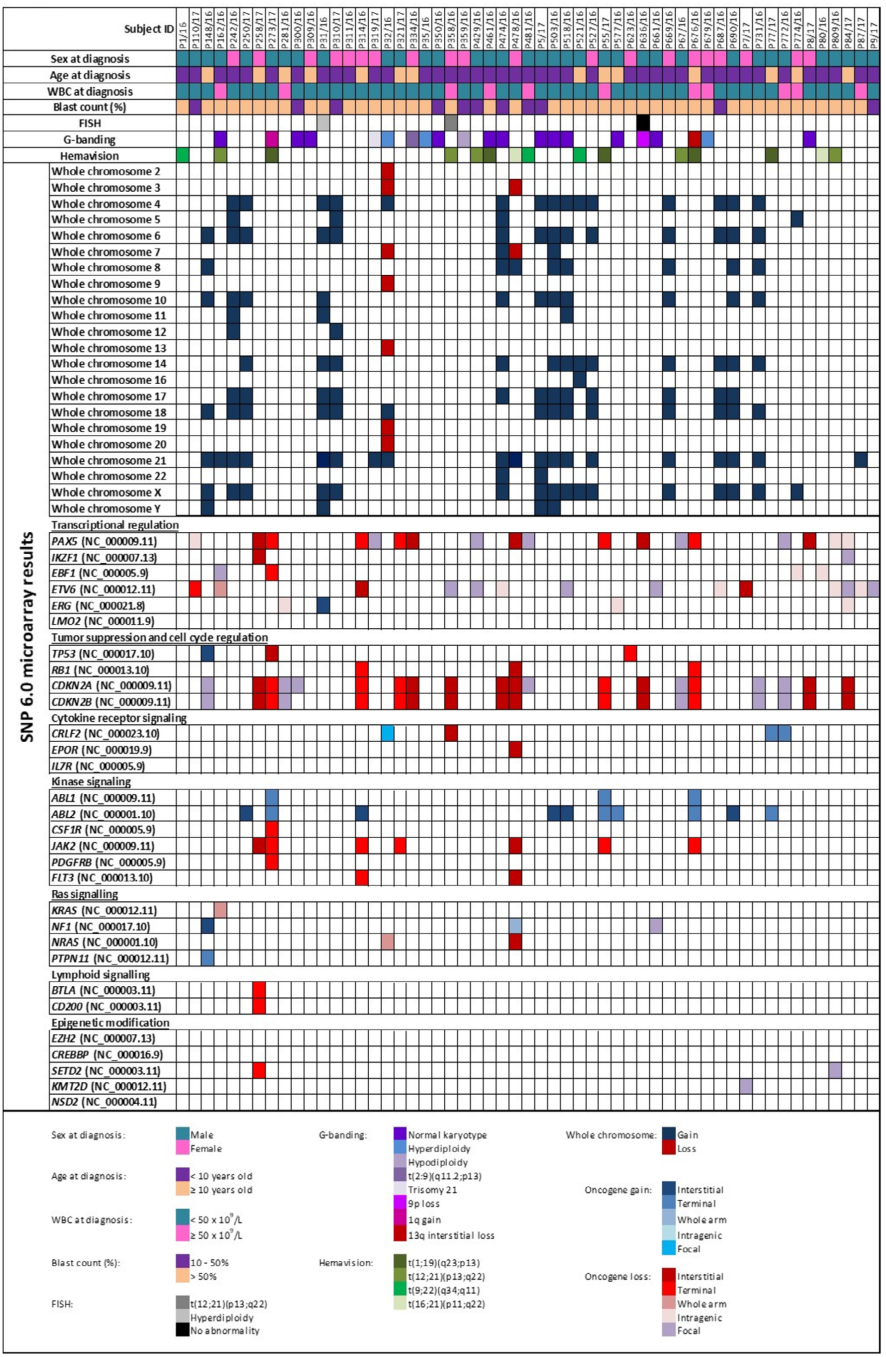
Mutational profile of 55 Malaysian childhood BCP‐ALL patients, including cytogenetics and molecular abnormalities. Each column represents a patient sample, and the different rows highlight the abnormalities. The oncogenes are categorized into key pathways.

About 50 ng of genomic DNA was used for this experiment. Genome‐wide SNP array analysis was performed using the commercially available, pre‐designed Affymetrix Genome‐Wide Human SNP Sty/Nsp 6.0, which features 1.8 million genetic markers, including more than 906,600 single‐nucleotide polymorphisms (SNPs) and more than 946,000 probes for CNVs, following the manufacturer's instructions (Affymetrix, Santa Clara, CA). SNP array data were analysed using the Affymetrix ChAS software version 4.0.0.385 (Affymetrix). The plot of two parameters, log2 ratio and allele difference, was visually examined for copy‐number and genotype, respectively. The hg19 reference genome was used for the analysis. The filter was set to 50 markers for gain, 50 for loss, and no size limitations. The findings were compared with other databases, such as the Cataloge of Somatic Mutations in Cancer (COSMIC) (Tate et al. 2019) and the Toronto Database of Genomic Variants (DGV) (MacDonald et al. 2014) for the pathogenic genes in BCP‐ALL and common chromosomal variants in populations, respectively. The list of BCP‐ALL‐related genes was taken from COSMIC and compared with the SNP 6.0 microarray findings (Figure 1). Common population variants were excluded from the analysis (Table S2). The UCSC Genome Browser (Perez et al. 2024) was used to view the CNAs and potential disease‐causing genes. The analysis followed the recommendations of the American College of Medical Genetics and Genomics (ACMG) Technical Standard for chromosomal microarray analysis and interpreting CNAs in neoplastic disorders (Mikhail et al. 2019; Shao et al. 2021).

### 2.6 MLPA Analysis

Two different Multiplex Ligation‐dependent Probe Amplification (MLPA) probe mixes, P335 and P327 (MRC‐Holland, Amsterdam, NL), were used to detect the gain or loss of genes that are known to be implicated in B‐ALL. About 50 ng of DNA was used for the experiment. The protocol was performed according to the manufacturer's instructions. Data were analysed using Coffalyser.Net version 220513.1739 (MRC‐Holland) for copy‐number evaluation. Cut‐off values of < 0.7 and > 1.3 were used to define losses and gains, respectively.

### 2.7 Statistical Analysis

Baseline characteristics were summarized as percentages or means with standard deviations. Data analysis was done using IBM SPSS Statistics version 29.0.2.0 (IBM Corp 2023).

## 3 Results

### 3.1 Characteristics of BCP‐ALL Patients

Of the 55 patients, 63.6% (*n* = 35) were male, and 36.4% (*n* = 20) were female. The mean age was 6.4 years (range 1–16 years; SD = 4.39). In terms of ethnicity, 27 were Malays (49.1%), nine were Chinese (16.4%), one was Indian (1.8%), and 18 were of other ethnicities (32.7%). The mean white blood cell (WBC) count was 31.5 × 10^9^/l (range 1.4–160.5 × 10^9^/l; SD = 36.0), and the mean blast count was 68.4% (range 20%–97%; SD = 24.5).

### 3.2 Trypsin‐Leishman's Banding, RT‐PCR, and FISH Analyses

Trypsin‐Leishman's banding karyotyping analysis produced analysable chromosomes in 21 samples (38.1%) (Table 1). In contrast, 23 samples (41.8%) were not analysable, and 11 (20.0%) yielded inconclusive results, likely due to insufficient samples. Of the 21 analysable cases, 12 had a normal karyotype, three had hyperdiploidy, one had hypodiploidy, and the remaining exhibited specific abnormalities; each with t(2;9)(q11.2;p13), trisomy 21 with additional material of unknown origin at chromosome 7p22, interstitial loss at chromosome 13q13‐q14, 9p deletion, and 1q gain.

**TABLE 1:** Comparison of Trypsin‐Leishman's banding, RT‐PCR, and SNP 6.0 microarray karyotype in 21 BCP‐ALL samples.

Subject ID	Trypsin‐Leishman's banding	RT‐PCR	SNP 6.0 microarray karyotpye
P162/16	Normal	t(12;21)(p13;q22)	Trisomy 21
P273/17	1q gain	t(1;19)(q23;p13)	1q gain Terminal deletion at 5q, 9p, 17p, 19p
P300/16	Normal	No amplification	Normal
P309/16	Normal	No amplification	Normal
P319/17	Trisomy 21	No amplification	Trisomy 21
P32/16	Hyperdiploidy	No amplification	Hypodiploidy
P334/16	t(2;9)(q11.2;p13)	No amplification	9p deletion
P35/16	Hyperdiploidy	No amplification	Normal
P350/16	Normal	No amplification	Normal
P359/16	Hypodiploidy	No amplification	Normal
P461/16	Normal	t(1;19)(q23.3;p13)	Normal
P474/16	Normal	No amplification	High hyperdiploidy
P5/17	Normal	No amplification	Hyperdiploidy
P503/16	Normal	No amplification	High hyperdiploidy
P518/16	Normal	No amplification	High hyperdiploidy
P577/16	Normal	No amplification	1q gain
P636/17	9p deletion	No amplification	9p deletion
P661/16	Normal	No amplification	Normal
P676/16	13q deletion	t(1;19)(q23.3;p13)	13q deletion
P679/16	Hyperdiploidy	No amplification	Trisomy 21
P8/17	Normal	No amplification	Normal

RT‐PCR was used as part of the diagnostic workup, and gene rearrangement was identified in 15 samples (27.2%) including t(1;19)(q23;p13) (*n* = 5), t(12;21)(p13;q22) (*n* = 5), t(9;22)(q34;q11) (*n* = 3), and t(16;21)(p11;q22) (*n* = 2). However, only 20% of the RT‐PCR results concorded with the Trypsin‐Leishman's banding (Table 1). In addition, FISH analysis was performed on only three samples due to the restricted sample volumes received. One sample (P358/16) revealed t(12;21)(p13;q22), another (P31/16) showed hyperdiploidy, and the third sample (P636/16) displayed no aberration. A more detailed overview of all abnormalities in each patient is visualized in Figure 1.

### 3.3 SNP 6.0 Microarray Analyses

A total of 191 copy‐number abnormalities were identified by the SNP 6.0 microarray across all 55 patients. The analysis revealed 14 hyperdiploidy cases (*n* = 14/55, 25.4%) with extra copies of chromosomes ranging from 48 to 58 (Figure 1). Additionally, three patients (*n* = 3/55, 5.4%) had an extra copy of chromosome 21 and were diagnosed as Down Syndrome‐associated ALL. Two patients (*n* = 2/55, 3.6%) exhibited hypodiploidy, while the remaining patients (*n* = 36/55, 65.6%) had normal karyotypes but with detectable CNAs. Some CNAs contained genes that were involved in the pathogenicity of BCP‐ALL. Comparison with Trypsin‐Leishman's banding karyotyping results revealed concordance in 11 of 21 analysable cases (52.4%) (Table 1). In one instance, the unbalanced translocation t(2;9)(q11.2;p13) observed in the Trypsin‐Leishman's banding karyotyping was identified by SNP 6.0 microarray as a 9p13 deletion (Table 1). The SNP 6.0 microarray also detected hyperdiploidy and hypodiploidy in undetected cases by karyotyping (*n* = 4, 19.0%). The SNP 6.0 microarray also detected four whole‐arm abnormalities. Two whole‐arm gains were identified in a single patient (P478/16) involving the following regions: 14q11.1‐q32.33 (arr[hg19] 14q11.1q32.33(19,002,112‐107,285,437)×3) (87.7 Mb) and 17p11.2‐q25.3 (arr[hg19] 17p11.2q25.3(21,739,052‐80,765,322)×3) (59.0 Mb). Chromosomal losses were observed in one patient (P162/16), affecting specific regions 12p13.33‐p11.21 (arr[hg19] 12p13.33p11.21(334,452‐33,252,166)×1) (32.9 Mb), which include the oncogene *KRAS* (OMIM:190070), and in another patient (P32/16) at the region 1p36.33‐p11.2 (arr[hg19] 1p36.33p11.2(768,780‐121,343,784)×1) (120.6 Mb), which impacts oncogene *NRAS* (OMIM:164790).

#### 3.3.1 Interstitial Gains and Losses

Further analysis with the SNP 6.0 microarray revealed approximately 44 interstitial abnormalities across 28 patients. These included 12 interstitial gains in 11 patients, ranging in size from 2.9 Mb to 100.1 Mb, and 32 interstitial losses in 19 patients, ranging from 6.4 Mb to 122.7 Mb. Of 28 patients, two had both gain and loss (P273/17 and P314/16) (Figure 1). Pathogenic genes identified within the interstitial losses included *CDKN2A* (OMIM:600160) (*n* = 8/55, 14.5%), *CDKN2B* (OMIM:600431) (14.5%), *PAX5* (OMIM:167414) (9.1%), *ETV6* (OMIM:600618) (3.6%), *JAK2* (OMIM:147796) (3.6%), *CRLF2* (OMIM:300357) (1.8%), *EPOR* (OMIM:133171) (1.8%), *IKZF1* (OMIM:603023) (1.8%), *NRAS* (OMIM:164790) (1.8%), *RB1* (OMIM:614041) (1.8%), and *TP53* (OMIM:191170) (1.8%) (Figure 1). One patient (P478/16) had concurrent interstitial losses at multiple chromosomal regions, including 1p, 9p, 9q, 13q, 15q, 19p, 19q, 20p, 20q, and a rare translocation t(16;21)(p11;q22). Another patient (P358/16) exhibited a pseudoautosomal region 1 (PAR1) loss at chromosome Xp22.33, with the loss also extending across 122.7 Mb, encompassing the region Xp22.33‐q25 (arr[hg19] Xp22.33q25(400,108‐123,119,337)×1).

#### 3.3.2 Terminal Gains and Losses

In addition, 33 terminal abnormalities were identified in 15 patients, comprising 14 terminal gains and 19 terminal losses. Among these, four patients (P273/17, P55/17, P676/16, and P77/17) with terminal gains at chromosome 1q23.3‐q44 involving the *PBX1* gene (OMIM:176310) were found to have translocation t(1;19)(q23;p13)(*TCF/PBX1*) identified through RT‐PCR, but not by karyotyping and FISH. However, another patient (P461/16) with the same translocation detected by RT‐PCR exhibited no chromosomal abnormalities detected by the SNP 6.0 microarray.

#### 3.3.3 Intrachromosomal Amplification of Chromosome 21 (iAMP21)

One significant subtype in BCP‐ALL is iAMP21, which involves chromothripsis. Chromothripsis is a copy‐number profile with alternating copy‐number states in a single chromosome arm containing at least ten distinct copy‐number segments (Mikhail et al. 2019). Surprisingly, neither the SNP 6.0 microarray nor the MLPA analyses detected any instances of chromothripsis in our 55 patients (Table S3). All detections involved the amplification of chromosome 21.

#### 3.3.4 Focal Gains and Losses

**FIGURE 2:**
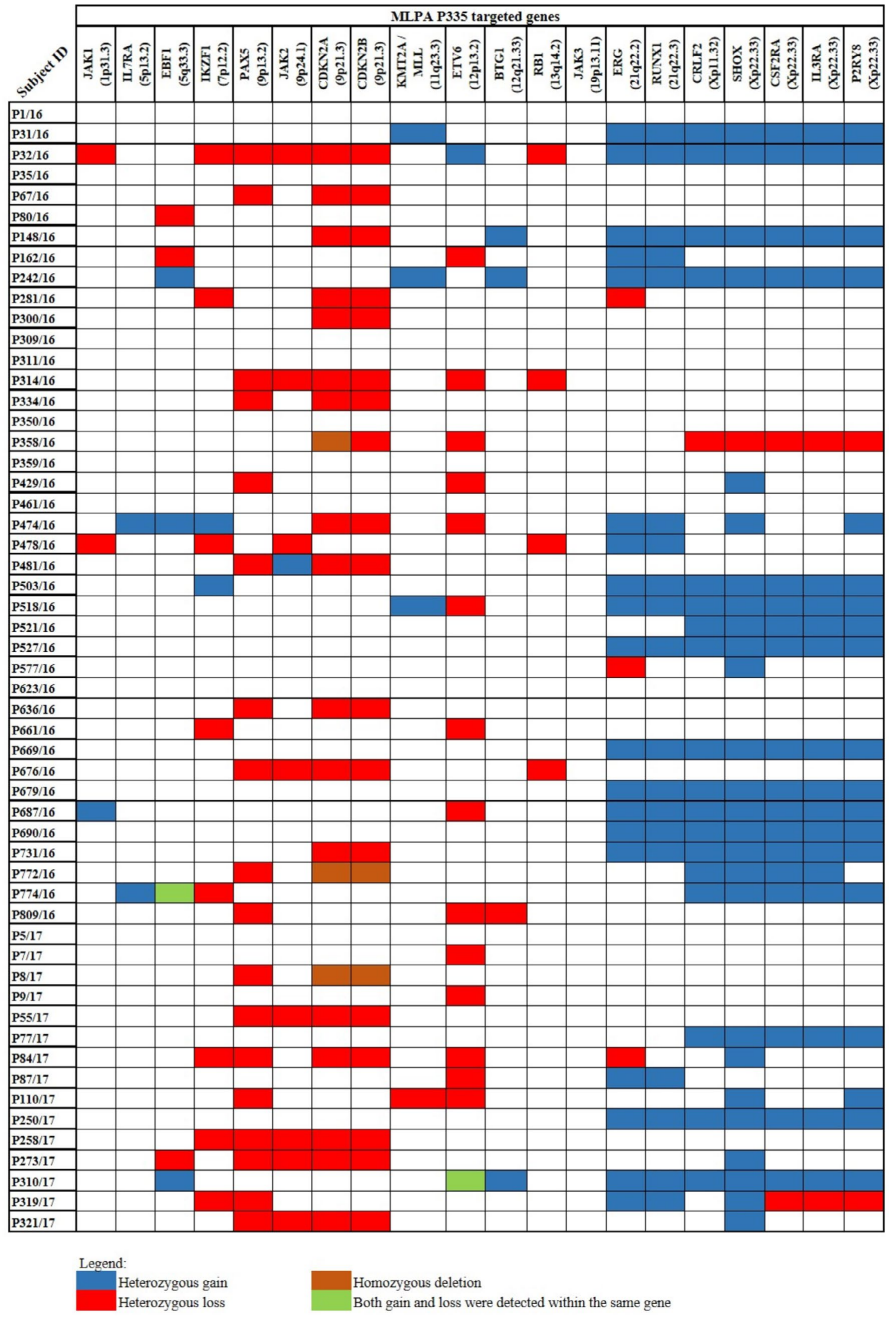
The MLPA P335 analysis of 55 Malaysian childhood BCP‐ALL patients revealed multiple gains and losses involving 20 pathogenic genes to BCP‐ALL.

Focal copy‐number abnormalities are relatively small genomic alterations, typically spanning less than 5 Mb, and often encompass genes known or suspected to drive cancer development (Mikhail et al. 2019). The SNP 6.0 microarray detected 71 focal copy‐number abnormalities in 34 patients, involving 55 focal losses (ranging in size from 69 to 3432 kb) and 13 focal gains (ranging from 81 to 3888 kb). The pathogenic genes involved in focal loss included *CDKN2A* (*n* = 7/55, 12.7%), *ETV6* (10.9%), *PAX5* (9.1%), *CDKN2B* (5.4%), *BTG1* (OMIM:109580) (1.8%), *EBF1* (OMIM:164343) (1.8%), *IKZF1* (1.8%), *KMT2D* (OMIM:602113) (1.8%), *NF1* (OMIM:613113) (1.8%), and *SETD2* (OMIM:612778) (1.8%) (Figure 1). MLPA results confirmed the focal loss of these genes (Figure 2). While focal gains in our study cohort involved some disease‐causing genes such as *SHOX*, *NYX*, *KRT2*, *PRKCD*, *TKT*, *MAPT*, *NSF*, and *KANSL1*, their involvement in the pathogenicity of BCP‐ALL remains uncertain. SNP 6.0 microarray also detected a region of focal loss (95.5 Mb) in a male patient (P5/17) involving the *HBB* gene.

#### 3.3.5 Intragenic Losses

Intragenic abnormalities involve the changes that occur in a single gene (Mikhail et al. 2019). The SNP 6.0 microarray detected 18 intragenic losses in 13 patients. Among the genes involved were *DPP6* (OMIM:126141) (*n* = 3/55, 5.4%), *EBF1* (3.6%), *ERG* (OMIM:165080) (5.4%), *ETV6* (5.4%), *IKZF3* (OMIM:606221) (5.4%), *MACROD2* (OMIM:611567) (5.4%), *NPHP4* (OMIM:607215) (5.4%), *PAX5* (5.4%), *SPTBN1* (OMIM:182790) (5.4%), and *WWOX* (OMIM:605131) (5.4%). The MLPA findings concorded with the loss of *EBF1*, *ERG*, *ETV6*, and *PAX5* (Figure 2).

## 4 Discussion

This study comprehensively analyses copy‐number abnormalities (CNAs) in a cohort of 55 Malaysian childhood BCP‐ALL patients utilizing SNP 6.0 microarray and complemented by MLPA to validate key oncogenes. Our findings revealed a diverse spectrum of cytogenetic and CNAs with potential implications for risk stratification and understanding disease pathogenesis. They emphasized the high‐resolution of microarray in detecting submicroscopic abnormalities missed by conventional karyotyping and showed the genetic heterogeneity of BCP‐ALL in this cohort. We also compared the findings with available Trypsin‐Leishman's banding, FISH, and RT‐PCR results from the diagnostic reports.

The SNP 6.0 microarray notably identified hyperdiploidy in 19% of cases undetected by karyotyping (Table 1), consistent with prior studies showing the enhancement of microarray sensitivity for aneuploidy and structural rearrangements (Mullighan et al. 2007). The discordance in P162/16, P32/16, P35/16, P359/16, P474/16, P5/17, P503/16, P518/16, P577/16, and P679/16 may reflect technical limitations of traditional cytogenetic methods and underscores the clinical utility of high‐resolution genomic profiling in BCP‐ALL diagnostics. The SNP 6.0 microarray also resolved ambiguous findings (e.g., reclassifying t(2;9) as a 9p deletion), emphasizing its role in defining diagnostic accuracy.

The comparison of SNP 6.0 microarray with conventional karyotyping revealed the superior resolution of the microarray in detecting CNAs, particularly submicroscopic and cryptic alterations. Our analysis detected a high frequency of CNAs (191 across 55 patients), emphasizing the vital role of genomic alterations in the pathogenesis and progression of BCP‐ALL. The detection of hyperdiploidy (25.4%) and Down syndrome‐associated ALL (5.4%) aligns with established knowledge of common cytogenetic subtypes in childhood BCP‐ALL (Haas and Borkhardt 2022; Maloney et al. 2010). The lower incidence of hypodiploidy (3.6%) in our cohort, although in agreement with the prevalence of hypodiploidy of BCP‐ALL (Molina et al. 2021), warrants further investigation in more extensive studies to determine if this is a genuine population‐specific characteristic.

Identifying submicroscopic abnormalities, including whole‐arm, interstitial, terminal, and focal CNAs, provides a more detailed understanding of the genomic aberrations in this cohort. The identification of recurrent interstitial and focal losses involving tumor suppressor genes and oncogenes, such as *CDKN2A/B*, *PAX5*, *ETV6*, *JAK2*, *CRLF2*, *EPOR*, *IKZF1*, *NRAS*, *RB1*, and *TP53*, emphasizes their established roles in BCP‐ALL pathogenesis (Lejman et al. 2022). The frequent loss of two tumor suppressor genes, *CDKN2A/B* (14.5%), located adjacent at chromosome 9p21, which encodes three proteins (p16INK4A, p14ARF, and p15INK4B) that belong to the *RB1* and *TP53* pathways, is particularly significant, as deletions in this locus are known to be associated with poor prognosis in some BCP‐ALL subtypes (Ampatzidou et al. 2023; Carrasco Salas et al. 2016; Kathiravan et al. 2019). Specifically, p16INK4A and p15INK4B act by inhibiting the proliferative kinases CDK4/CDK6, while p14ARF stabilizes p53, collectively preventing uncontrolled cell division and maintaining normal cellular growth. Inactivation or deletion of *CDKN2A/B* genes removes these critical cell cycle brakes, leading to rapid and uncontrolled cell proliferation, a hallmark characteristic of cancer formation (Črepinšek et al. 2024, 5; Shahjahani et al. 2015). Similarly, the recurrent involvement of an oncogene, *PAX5* (9.1%), and a tumor‐suppressor gene, *ETV6* (3.6%), the key genes for B‐cell development, further emphasizes their importance in leukemogenesis. The Pax5 transcription factor, also known as B‐cell‐specific activator protein (BSAP), plays a pivotal role in the hematopoietic system. Pax5 activates the expression of factors required for the (Pre) BCR signaling pathway. The deletion of the *PAX5* gene causes cell arrest in the Pro‐B‐cell stage (Črepinšek et al. 2024, 5; Shahjahani et al. 2015). Patients having translocation involving the *PAX5* gene did not respond well to the treatment (Chang et al. 2024). Moreover, in this study, a patient (P478/16) with concurrent interstitial losses involving pathogenic genes and rare t(16;21) translocation remarkably achieved remission with the BFM95 treatment protocol (Table S1). We also identified a patient (P273/17) with concurrent interstitial gains and losses involving pathogenic genes. The patient responded well to treatment despite the co‐occurrence of interstitial gains and losses (Table S1).

Detecting terminal abnormalities, including gains and losses, and their correlation with *PBX1* rearrangement in t(1;19)(q23;p13) positive cases accentuates the complex interaction between different types of genomic alterations in childhood BCP‐ALL. Interestingly, the discrepancy observed in one t(1;19)‐positive case, where the SNP 6.0 microarray detected no CNAs, suggests potential limitations in detecting balanced translocations or the presence of breakpoints outside the coverage of the CNA probes. This highlights the importance of integrating different diagnostic modalities, including RT‐PCR and FISH, for a comprehensive genetic characterization. The *PBX1* rearrangement involves a fusion of the *E2A* gene (also known as *TCF3*) on chromosome 19 and the *PBX1* gene on chromosome 1 (Hu et al. 2016). The *E2A‐PBX1* fusion protein disrupts normal B‐cell development and is thought to contribute to the development of BCP‐ALL. This translocation is often associated with an intermediate prognosis (Malhotra et al. 2022).

A key finding was the absence of iAMP21 in our cohort. Patients with iAMP21 were reported to have a higher risk of relapse and worse overall survival compared to other patients with ALL (Harrison 2015). The SNP 6.0 microarray and MLPA detected no iAMP21 in our cohort, suggesting a potentially lower prevalence of this specific high‐risk feature in Malaysian childhood BCP‐ALL compared to some other populations. Wang et al. (2016) reported iAMP21 in 5% of 60 B‐ALL cases in the United States (Wang et al. 2016), while Hormann et al. (2023) detected iAMP21 in 7.7% of 155 BCP‐ALL cases in the Netherlands (Hormann et al. 2023). However, given the sample size, further studies with larger cohorts are needed to establish the frequency of iAMP21 in the Malaysian population.

The high number of focal CNAs detected by the SNP 6.0 microarray highlights the presence of small but potentially functionally significant genomic alterations in our cohort. While some of the genes involved in focal losses, such as *CDKN2A*, *ETV6*, *PAX5*, *CDKN2B*, *BTG1*, *EBF1*, *IKZF1*, *KMT2D*, *NF1*, and *SETD2*, are known to be relevant in BCP‐ALL, the clinical significance of the genes involved in focal gains in our cohort requires further investigation. The SNP 6.0 microarray detected a patient (P5/17) with focal loss of the *HBB* gene. However, this patient was not reported to have symptoms of beta‐thalassemia. Loss of the *HBB* gene has been reported in acute myeloid leukemia (Luo et al. 2022), but no studies have yet documented its role in BCP‐ALL. Interestingly, the *IKZF1* loss, which is associated with poor prognosis in BCP‐ALL, is more prevalent in other populations (Stanulla et al. 2020; Wang et al. 2016). Still, the very low frequency in our cohort suggests that this mutation is possibly rare in our population (Figure 1). *IKZF1* is the gene that encodes the IKAROS protein, a crucial transcription factor involved in the development of lymphocytes (Crepinsek et al. 2021). Loss of IKZF1 disrupts the normal function of IKAROS, contributing to the development and progression of leukemia (Paolino et al. 2024).

The identification of intragenic losses in several genes, including *DPP6*, *EBF1*, *ERG*, *ETV6*, *IKZF3*, *MACROD2*, *NPHP4*, *PAX5*, *SPTBN1*, and *WWOX*, further expands the spectrum of genomic abnormalities observed in Malaysian childhood BCP‐ALL. The concordance of MLPA results with the loss of *EBF1*, *ERG*, *ETV6*, and *PAX5* validates the microarray findings. Intragenic *ERG* loss often occurs recurrently with *DUX4*‐rearrangement (Zaliova et al. 2019). However, in this study, the mutation was detected without the *DUX4*‐rearrangement. Intragenic *ERG* loss was found in 3.2% of childhood BCP‐ALL and had a favorable treatment outcome (Clappier et al. 2014). The potential functional consequences of these intragenic losses warrant further investigation at the RNA and protein levels.

The identification of specific CNAs, such as recurrent interstitial and focal losses involving tumor suppressor genes and oncogenes, allows for more accurate risk stratification of BCP‐ALL patients (Azeez et al. 2025; Østergaard and Iacobucci 2025). Besides that, the varied responses observed in patients with concurrent genomic aberrations highlight that treatment outcomes are not solely determined by individual genetic abnormalities. This emphasizes the importance of considering the entire genomic and biological context to tailor individualized, risk‐adapted treatment regimens. The ability to identify specific genetic subtypes and their associated prognosis allows for the application of more precise therapeutic strategies, ultimately leading to improved clinical outcomes (Ratti et al. 2020; Ray et al. 2024). Moreover, understanding the molecular mechanisms by which genomic alterations activate oncogenic pathways can help in designing novel therapies to overcome drug resistance.

There were some limitations in this study. The modest cohort size (*n* = 55) limits statistical power for subgroup analyses (e.g., oncogene‐specific or ethnicity‐specific CNAs). Additionally, longitudinal data linking CNAs to outcomes is needed. Also, SNP microarray cannot detect balanced chromosomal rearrangements, including translocations. These structural variations, particularly those involving genes implicated in key signaling pathways, are frequently observed in childhood BCP‐ALL and have substantial prognostic significance. To overcome this limitation, a complementary approach using a combined SNP microarray with FISH or RNA sequencing for fusion gene analysis can effectively identify these clinically relevant genetic alterations (Berry et al. 2019; Gourmel et al. 2024). Due to the unavailability of normal samples, we cannot completely rule out the possibility that some of the identified abnormalities may represent germline mutations rather than somatic mutations, despite rigorous screening against COSMIC and DGV databases.

## 5 Conclusion

In conclusion, SNP 6.0 microarray and MLPA analyses increased the detection of some unique copy‐number abnormalities in the Malaysian BCP‐ALL cohort compared to other methods. Taken together, the SNP 6.0 microarray and MLPA provide valuable insights into the genetic landscape of this disease in the Malaysian population. Incorporating these techniques as diagnostic tools will improve the understanding of the genetic landscape of Malaysian childhood BCP‐ALL, facilitate treatment stratification, and improve patient care management.

## Author Contributions

N.S.M.D. performed the SNP 6.0 microarray analysis and wrote the first draft of the manuscript. N.A.A. performed the genomic DNA extraction and MLPA analysis. M.F.Z. and Z.Z. performed the FISH analysis. D.A.Z., E.E., and Y.M.Y. performed the Trypsin‐Leishman's banding karyotyping analysis. N.R.K. performed the RT‐PCR analysis. S.‐L.Y., B.L.‐S.H., N.D.D., A.S.A.G., and W.A.H.W.H. contributed the samples and clinical data. All authors have reviewed the manuscript.

## Funding

This study was funded by the Malaysian Ministry of Health (MOH) grant, NMRR‐16‐1468‐32133.

## Ethics Statement

This study was approved by the Malaysian Research and Ethical Committee (MREC), NMRR‐16‐1468‐32133 (IIR).

## Conflicts of Interest

The authors declare no conflicts of interest.

## Supporting information


**Data S1:** Supplementary Methods.

**Table S1:** Clinical parameters for 21 patients with BCP‐ALL, including risk group, treatment protocol, and treatment outcome.

**Table S2:** Common gain and loss variants excluded from the analysis.

**Table S3:** SNP 6.0 microarray and MLPA P327 results of chromosome 21 in 55 Malaysian patients with BCP‐ALL. The results showed no chromothripsis occurred in any patients.

## Data Availability

Data are available upon request.
