# Supplementary material for: Single Nucleotide Polymorphism Microarray Analysis Unveils Copy‐Number Abnormalities and Genetic Heterogeneity in Malaysian Childhood B‐Cell Precursor Acute Lymphoblastic Leukemia
Source: Mol Genet Genomic Med. 2026 Mar 2;14(3):e70182. doi: 10.1002/mgg3.70182 (PMC12953716; doi:10.1002/mgg3.70182)
Supplement: Supplementary file 1 — Data S1: Supplementary Methods. [file MGG3-14-e70182-s004.docx]

**Supporting Information Methods**

Trypsin-Leishman’s Banding Karyotyping

Bone marrow cells were prepared by culturing the cells in RPMI 1640 medium supplemented with 20% fetal calf serum (GIBCO) at 37°C for 24 hours. During the final hour of incubation, the cultures were treated with 5 µg/mL colcemid. Following treatment, cells were harvested using standard procedures, including hypotonic shock with 0.075 M KCl, and were fixed in methanol:acetic acid (3:1) solution. The fixed cells were then dropped onto clean glass slides and allowed to air dry. A 0.08% Trypsin solution was prepared using 0.04 g trypsin powder dissolved in 50 ml phosphate buffer saline, pH 7.3. Leishman’s stain was prepared using 1 part stain diluted with 6 parts phosphate buffer, pH 6.8. The slides were dipped in PBS for 1 minute, followed by Trypsin for 5 – 10 seconds. The slides were rinsed in two Coplin jars containing PBS solutions. The cells were stained with fresh Leishman’s stain for four minutes, then rinsed under running water and air drying. The slides were viewed under a brightfield microscope for quality chromosome bands. A minimum of 20 metaphase cells were analyzed, and any chromosomal abnormalities were identified and named according to the International System for Human Cytogenetic Nomenclature (ISCN 2016)^1^.

Fluorescence In Situ Hybridization (FISH)

The B-ALL FISH panel consisted of the *ETV6(TEL)*/*RUNX1(AML1)* ES dual-color translocation probe (Vysis-Abbott Molecular Inc. Des Plaines, IL 60018, USA) to detect the t(12;21)(p13;q22) that results in the *ETV6/RUNX1* fusion, *KMT2A/MLL* (11q23.3) Dual Color Break Apart Rearrangement (Vysis-Abbott Molecular Inc.), and *CRLF2* (Xp22.33/Yp11.32) break-apart probe (Cytocell, Ltd., Cambridge, UK). Samples were processed for FISH analysis following the manufacturers’ recommendations. Briefly, the samples were prepared by air-drying cells onto microscope slides, which were subsequently washed in 2× SSC buffer for 2 minutes at room temperature. Dehydration was achieved by immersing the slides sequentially in an ethanol series of 70%, 85%, and 100%, each for 2 minutes. Following dehydration, 10 μL of the probe mixture was applied to the cell sample, and a cover slip was placed over the probe area, sealed with rubber solution glue to prevent evaporation. The slides were then incubated at 37°C for 16 hours to allow the probes to hybridize with complementary chromosomal sequences. After hybridization, unbound probes were removed through a series of washing steps with 0.4X SSC for 2 minutes, followed by 2X SSC for 30 seconds. The slides were counterstained with DAPI antifade solution (10 μL) to enhance nuclear visualization. Finally, FISH images were analyzed using an Olympus BX51 fluorescence microscope (Olympus, Tokyo, Japan) linked to the automated cytogenetics platform Cytovision^®^ version 7.3.1 (Leica Biosystem, Nussloch, Germany). At least two hundred analyzable interphase cells were scored for each probe by two different examiners with a cut-off value of 5%.

List of Translocations Detected by the Reverse Transcriptase-Polymerase Chain Reaction (RT-PCR)^2^

| del1(p32) (STIL-TAL1) | t(9;12) (q34;p13) (ETV6-ABL1) |
| --- | --- |
| t(1;11) (p32;q23) (MLL-EPS15) | t(9,22) (q34;q11) (BCR-ABL1) |
| t(1;11) (q21;q23) (MLL-MLLT11) | t(10;11) (p12;q23) (MLL-MLLT10) |
| t(1;19) (q23;p13) (TCF3-PBX1) | t(11;17) (q23;q21) (MLL-MLLT6) |
| t(3;5) (q25;q34) (NPM1-MLF1) | t(11;17) (q23;q21) (ZBTB16-RARA) |
| t(3;21) (q26;q22) (RUNX1-MECOM) | t(11;19) (q23;p13.1) (MLL-ELL) |
| t(4;11) (q21;q23) (MLL-AFF1) | t(11;19) (q23;p13.3) (MLL-MLLT1) |
| t(5;12) (q33;p13) (ETV6-PDGFRB) | t(12;21) (p13;q22) (ETV6-RUNX1) |
| t(5;17) (q35;q21) (NPM1-RARA) | t(12;22) (p13;q11) (ETV6-MN1) |
| t(6;9) (p23;q34) (DEK-NUP214) | t(15;17) (q24;q21) (PML-RARA) |
| t(6;11) (q27;q23) (MLL-MLLT4) | inv(16) (p13;q22) (CBFB-MYH11) |
| t(8;21) (q22;q22) (RUNX1-RUNX1T1) | t(16;21) (p11;q22) (FUS-ERG) |
| t(9;9) (q34;q34) (SET-NUP214) | t(17;19) (q22;p13) (TCF3-HLF) |
| t(9;11) (p22;q23) (MLL-MLLT3) | t(X;11) (q13;q23) (MLL-FOXO4) |

Reference:

[1] McGowan-Jordan J, Simons A, Schmid M. ISCN 2016. An international system for human cytogenomic nomenclature. Karger; 2016.

[2] HemaVision^®^-28N Multiplex RT-PCR Test Screens for 28 Leukemia Causing Translocations (Cat. No: HV01-28N), DNA Diagnostic A/S, Risskov, Denmark.
