# Supplementary material for: Single Nucleotide Polymorphism Microarray Analysis Unveils Copy‐Number Abnormalities and Genetic Heterogeneity in Malaysian Childhood B‐Cell Precursor Acute Lymphoblastic Leukemia
Source: Mol Genet Genomic Med. 2026 Mar 2;14(3):e70182. doi: 10.1002/mgg3.70182 (PMC12953716; doi:10.1002/mgg3.70182)
Supplement: Supplementary file 2 — Table S1: Clinical parameters for 21 patients with BCP‐ALL, including risk group, treatment protocol, and treatment outcome. [file MGG3-14-e70182-s001.docx]

Supporting Information Table 1: Clinical parameters for 21 patients with BCP-ALL, including risk group, treatment protocol, and treatment outcome.

| **Subject ID** | **Sex** | **Age at diagnosis** | **Platelet (x10^9^/L)** | **Hb (g/dL)** | **WBC count (x10^9^/L)** | **Blast count (%)** | **Diagnosis** | **Risk group*** | **Treatment protocol** | **Treatment outcome** |
| --- | --- | --- | --- | --- | --- | --- | --- | --- | --- | --- |
| P67/16 | Male | 3.8 | 83.0 | 11.8 | 30.33 | 65.0 | BCP-ALL | Medium | BFM 95 | Remission |
| P242/16 | Female | 1.6 | 47.0 | 6.0 | 29.0 | 60.0 | BCP-ALL | Standard | BFM 95 | Remission |
| P281/16 | Male | 14.0 | 40.0 | 4.3 | 92.6 | 94.0 | BCP-ALL | Medium | BFM 95 | Remission |
| P300/16 | Male | 8.6 | 168.0 | 9.1 | 1.4 | 24.0 | BCP-ALL | Medium | BFM 2002 | Remission |
| P429/16 | Male | 4.0 | 16.0 | 4.5 | 13.6 | 35.0 | BCP-ALL | Standard | BFM 2002 | Remission |
| P478/16 | Female | 5.0 | 104.0 | 5.9 | 5.9 | 90.0 | BCP-ALL | Standard | BFM 95 | Remission |
| P521/16 | Male | 14.0 | 13.0 | 11.7 | 1.75 | 90.0 | BCP-ALL | High | BFM 2002 | Death |
| P577/16 | Male | 10.0 | 51.0 | 5.5 | 17.6 | 97.0 | BCP-ALL | Standard | UKALL 2003 | Remission |
| P661/16 | Male | 9.0 | 56.0 | 2.5 | 26.3 | 78.0 | BCP-ALL | Standard | BFM 2009 | Remission |
| P669/16 | Female | 1.4 | 10.0 | 2.9 | 6.84 | 95.0 | BCP-ALL | Standard | BFM 95 | Remission |
| P676/16 | Female | 14.0 | 13.0 | 3.4 | 154.0 | 90.0 | BCP-ALL | High | BFM 2002 | Death |
| P774/16 | Female | 16.0 | 37.0 | 3.5 | 57.1 | 66.0 | BCP-ALL | High | BFM 2002 | Death |
| P809/16 | Male | 6.0 | 24.0 | 4.6 | 43.2 | 55.0 | BCP-ALL | Medium | BFM 95 | Remission |
| P7/17 | Female | 4.6 | 89.0 | 6.4 | 19.7 | 67.0 | BCP-ALL | Medium | BFM 95 | Remission |
| P8/17 | Female | 6.6 | 85.0 | 10.8 | 8.5 | 53.0 | BCP-ALL | Medium | BFM 95 | Remission |
| P9/17 | Male | 4.8 | 13.0 | 5.2 | 5.4 | 22.0 | BCP-ALL | Standard | BFM 95 | Remission |
| P55/17 | Male | 11.0 | 27.0 | 5.1 | 72.2 | 81.0 | BCP-ALL | Medium | BFM 95 | Remission |
| P84/17 | Male | 11.0 | 16.0 | 8.2 | 9.4 | 56.0 | BCP-ALL | Medium | BFM 95 | Remission |
| P258/17 | Female | 15.0 | 85.0 | 6.9 | 23.1 | 74.0 | BCP-ALL | Medium | BFM 95 | Remission |
| P273/17 | Male | 9.0 | 9.0 | 8.9 | 10.7 | 90.0 | BCP-ALL | Medium | BFM 2002 | Remission |
| P319/17 | Female | 4.0 | 134.0 | 6.4 | 29.1 | 90.0 | Down syndrome-ALL | Standard | BFM 2009 | Death |

*Based on the 2016 revision of the World Health Organization classification of lymphoid neoplasms (Swerdlow et al. 2016).
