# Supplementary material for: Single Nucleotide Polymorphism Microarray Analysis Unveils Copy‐Number Abnormalities and Genetic Heterogeneity in Malaysian Childhood B‐Cell Precursor Acute Lymphoblastic Leukemia
Source: Mol Genet Genomic Med. 2026 Mar 2;14(3):e70182. doi: 10.1002/mgg3.70182 (PMC12953716; doi:10.1002/mgg3.70182)
Supplement: Supplementary file 3 — Table S2: Common gain and loss variants excluded from the analysis. [file MGG3-14-e70182-s002.zip › MGG3_70182__author.pdf]

Supporting Information Table 2: Common gain and loss variants excluded from the analysis.

Sample ID	Age at diagnosis	Ethnicity	Gender	Gain					
				Chr.	Cytoband	Location	Size (kb)	Gene(s)	Nomenclature
P31/16	8.0	Malay	male	15	q11.1-q11.2	20175851-22311799	2129	CHEK2P2, HERC2P3, GOLGA6L6, GOLGA8CP, NBEAP1, MIR3118-2, MIR3118-4, MIR3118-3, POTEB, POTEB2, POTEB3, NF1P2, MIR5701-2, MIR5701-3, MIR5701-1, LINC01193, LINC02203, FAM30C, LOC646214, CXADRP2, NF1P2, LINC02203, LOC101927079, OR4M2, OR4N4, OR4N3P, LOC102724760, IGHV1OR15-1, LOC642131, IGHV1OR15-3, MIR1268A, REREP3	arr[hg19] 15q11.1q11.2(20,175,851-22,311,799)x3
				16	q22.2	70854380-71065311	211	HYDIN	arr[hg19] 16q22.2(70,854,380-71,065,311)x3
P478/16	5.0	Malay	female	1	p36.66	61722-356530	295	OR4F5, LOC729737, LOC100132287, LOC100133331, LOC100132062	arr[hg19] 1p36.66(61,722-356,530)x3
				4	q35.2	190822374-191020138	198	FRG1, FRG2, DUX2, DUX4	arr[hg19] 4q35.2(190,822,374-191,020,138)x3
				10	q11.21	42525111-42850847	326	LOC441666	arr[hg19] 10q11.21(42,525,111-42,850,847)x3
				12	p13.31	8393815-8559653	166	FAM86FP, LINC00937	arr[hg19] 12p13.31(8,393,815-8,559,653)x3
P518/16	7.0	Chinese	male	1	1q41	215184412-215605450	421	KCNK2	arr[hg19] 1q41(215,184,412-215,605,450)x3
				15	q11.1-q11.2	20175851-22042741	1856	CHEK2P2, HERC2P3, GOLGA6L6, GOLGA8CP, NBEAP1, MIR3118-3, MIR3118-2, MIR3118-4, POTEB, POTEB2, POTEB3, NF1P2, MIR5701-3, MIR5701-2, MIR5701-1, LINC01193, LINC02203, FAM30C	arr[hg19] 15q11.1q11.2(20,175,851-22,042,741)x3
				15	q11.2	25415167-25499916	85	SNHG14, SNORD115	arr[hg19] 15q11.2(25,415,167-25,499,916)x3
				22	q11.22	23112534-23268151	156	IGLL5	arr[hg19] 22q11.22(23,112,534-23,268,151)x3
P679/16	1.9	Malay	female	14	q32.33	106218572-107100597	843	FAM30A, ADAM6, LINC00226, LINC00221	arr[hg19] 14q32.33(106,218,572-107,100,597)x3
				22	q12.3	33421749-33726223	304	SYN3, LARGE1	arr[hg19] 22q12.3(33,421,749-33,726,223)x4
P148/16	10.6	Chinese	male	14	q32.33	106044357-106900753	856	FAM30A	arr[hg19] 14q32.33(106,044,357-106,900,753)x3

				15	q11.2	20820054-22539947	1720	NBEAP1, POTEB	arr[hg19] 15q11.2(20,820,054-22,539,947)x3
P309/16	12.0	Sino Dusun	female	5	p15.33	730978-886975	156	ZDHHC11B, ZDHHC11, BRD9	arr[hg19] 5p15.33(730,978-886,975)x3
				14	q32.33	106482115-106777331	295	LINC00226	arr[hg19] 14q32.33(106,482,115-106,777,331)x4
P334/16	12.0	Malay	female	11	q14.1	81474348-82106888	633	MIR4300HG	arr[hg19] 11q14.1(81,474,348-82,106,888)x3
P358/16	5.0	Chinese	female	15	q13.3	32008984-32419762	411	OTUD7A, CHRNA7	arr[hg19] 15q13.3(32,008,984-32,419,762)x3
				22	q11.22	22997798-23259859	262	IGLL5	arr[hg19] 22q11.22(22,997,798-23,259,859)x3
P636/16	8.0	Bajau	male	22	q11.23-q12.1	25663972-25918709	255	LRP5L, CRYBB2P1, MIR6817	arr[hg19] 22q11.23q12.1(25,663,972-25,918,709)x3
P676/16	14.0	Malay	female	22	q11.2-q12.1	25669568-25911436	242	LRP5L, CRYBB2P1, MIR6817	arr[hg19] 22q11.23q12.1(25,669,568-25,911,436)x3
P772/16	4.0	Malay	male	22	q11.22	23090866-23275341	184	IGLL5	arr[hg19] 22q11.22(23,090,866-23,275,341)x3
P521/16	14.0	Malay	male	22	q11.22	23027410-23275341	248	IGLL5	arr[hg19] 22q11.22(23,027,410-23,275,341)x3
P1/16	2.0	Malay	male	14	q32.33	106194464-106923069	729	FAM30A	arr[hg19] 14q32.33(106,194,464-106,923,069)x3
				14	q11.2	19437974-20422583	985	POTEG	arr[hg19] 14q11.2(19,437,974-20,422,583)x3
				15	q11.1-q11.2	20590014-22673387	2083	NBEAP1, POTEB	arr[hg19] 15q11.1q11.2(20,590,014-22,673,387)x4
P32/16	1.8	Chinese	male	14	q32.33	106119626-107059052	939	FAM30A	arr[hg19] 14q32.33(106,119,626-107,059,052)x3
				22	q11.22	22752667-23275341	523	PRAME, GGTLC2, MIR650	arr[hg19] 22q11.22(22,752,667-23,275,341)x3
P35/16	4.0	Chinese	male	1	q31.3	196744123-196812518	68	CHFR3. CHFR1	arr[hg19] 1q31.3(196,744,123-196,812,518)x3
				13	q14.11	43632976-43929302	296	DNAJC15, ENOX1	arr[hg19] 13q14.11(43,632,976-43,929,302)x3
				14	q32.33	106197114-106927570	730	FAM30A	arr[hg19] 14q32.33(106,197,114-106,927,570)x4
				15	q11.2	25416713-25502419	86	SNHG14	arr[hg19] 15q11.2(25,416,713-25,502,419)x3
				22	q11.22	23040137-23268562	228	IGLL5	arr[hg19] 22q11.22(23,040,137-23,268,562)x3

P80/16	1.9	Chinese	male	15	q11.1-q11.2	20262223-22539947	2278	NBEAP1, POTEB	arr[hg19] 15q11.1q11.2(20,262,223-22,539,947)x3
				22	q11.22	23046122-23275341	229	IGLL5	arr[hg19] 22q11.22(23,046,122-23,275,341)x3
P162/16	5.6	Malay	male	15	q11.1-q11.2	20265965-22588019	2322	NBEAP1, POTEB	arr[hg19] 15q11.1q11.2(20,265,965-22,588,019)x3
				22	q11.22	23023184-23271342	248	IGLL5	arr[hg19] 22q11.22(23,023,184-23,271,342)x3
P242/16	1.6	Malay	female	9	p21.2	27154616-27249124	95	TEK	arr[hg19] 9p21.2(27,154,616-27,249,124)x3
P300/16	8.6	Malay	male	3	p21.31-p21.2	50431193-50617579	186	CACNA2D2	arr[hg19] 3p21.31p21.2(50,431,193-50,617,579)x3
				9	q34.2	137163661-137375773	212	RXRA	arr[hg19] 9q34.2(137,163,661-137,375,773)x3
P311/16	3.6	Kadazan	female	1	q31.3	196731147-196816718	86	CFHR3, CFHR1	arr[hg19] 1q31.3(196,731,147-196,816,718)x3
P281/16	14.0	Melanau	male	4	q13.2	69375335-69485967	111	UGT2B17	arr[hg19] 4q13.2(69,375,335-69,485,967)x1
P359/16	1.0	Iban	female	10	q11.21	43588195-43645854	58	RET, CSGALNACT2	arr[hg19] 10q11.21(43,588,195-43,645,854)x3
				15	q11.2	25416713-25499916	83	SNHG14	arr[hg19] 15q11.2(25,416,713-25,499,916)x3
P429/16	4.0	Malay	male	5	p15.33	453637-670745	217	EXOC3, SLC9A3, CEP72, TPPP	arr[hg19] 5p15.33(453,637-670,745)x3
				7	p12.2	49293919-49870692	577	VWC2	arr[hg19] 7p12.2(49,293,919-49,870,692)x3
P461/06	2.9	Malay	male	1	q31.3	196711066-196816718	106	CFH, CFHR3, CFHR1	arr[hg19] 1q31.3(196,711,066-196,816,718)x3
				14	q32.33	106218572-106922519	704	FAM30A	arr[hg19] 14q32.33(106,218,572-106,922,519)x3
P474/16	6.0	Malay	male	15	q11.2	25416713-25497305	81	SNHG14	arr[hg19] 15q11.2(25,416,713-25,497,305)x3
				22	q11.22	22292994-22579483	286	TOP3B	arr[hg19] 22q11.22(22,292,994-22,579,483)x3
				22	q11.22	23035011-23268562	234	IGLL5	arr[hg19] 22q11.22(23,035,011-23,268,562)x3
P481/16	1.0	Bugis	male	5	p15.31	8465019-9160472	695	SEMA5A, LINC02199	arr[hg19] 5p15.31(8,465,019-9,160,472)x3
				14	q32.33	106223847-106440472	217	FAM30A	arr[hg19] 14q32.33(106,223,847-106,440,472)x3

				15	q11.2	25416713-25489376	73	SNHG14	arr[hg19] 15q11.2(25,416,713-25,489,376)x3
P661/16	9.0	Malay	male	15	q11.1-q11.2	20586646-22042741	1456	NBEAP1, POTEB	arr[hg19] 15q11.1q11.2(20,586,646-22,042,741)x3
				22	q11.22	22905392-23275341	370	GGTLC2, IGLL5, MIR650	arr[hg19] 22q11.22(22,905,392-23,275,341)x3
P809/16	6.0	Bidayuh	male	15	q11.1-q11.2	20586646-22588019	2001	NBEAP1, POTEB	arr[hg19] 15q11.1q11.2(20,586,646-22,588,019)x3
				22	q11.22	23040137-23268562	228	IGLL5, MIR650	arr[hg19] 22q11.22(23,040,137-23,268,562)x3
P774/16	16.0	Chinese	female	22	q11.22	23040137-23268562	228	IGLL5, MIR650	arr[hg19] 22q11.22(23,040,137-23,268,562)x3
P503/16	2.0	Malay	male	22	q11.22	23027410-23275341	248	IGLL5	arr[hg19] 22q11.22(23,027,410-23,275,341)x3
P5/17	2.1	Sungai	male	14	q32.33	106224341-107285437	1061	FAM30A	arr[hg19] 14q32.33(106,224,341-107,285,437)x4
				19	q13.31	43559329-43671903	113	PSG2	arr[hg19] 19q13.31(43,559,329-43,671,903)x3
P7/17	4.6	Indian	female	1	p35.3-p35.2	29844092-30747564	903	LINC01648	arr[hg19] 1p35.3p35.2(29,844,092-30,747,564)x3
				14	q32.33	106218572-106918082	700	FAM30A	arr[hg19] 14q32.33(106,218,572-106,918,082)x3
				16	q23.3-q44	84044699-84098615	54	SLC38A8, MBTPS1	arr[hg19] 16q23.3(84,044,699-84,098,615)x3
				17	q21.31	44165802-44364158	198	KANSL1	arr[hg19] 17q21.31(44,165,802-44,364,158)x3
				22	q11.22	22905392-23268151	363	GGTLC2, IGLL5, MIR650	arr[hg19] 22q11.22(22,905,392-23,268,151)x3
				X	q28	154735697-154997107	261	TMLHE, SPRY3	arr[hg19] Xq28(154,735,697-154,997,107)x3
P8/17	6.6	Iban	female	7	p21.2	15606337-15866901	261	MEOX2	arr[hg19] 7p21.2(15,606,337-15,866,901)x3
				14	q32.33	106246289-106918637	672	FAM30A	arr[hg19] 14q32.33(106,246,289-106,918,637)x3
P55/17	11.0	Chinese	male	14	q32.33	106218572-106320374	102	Intron	arr[hg19] 14q32.33(106,218,572-106,320,374)x3
				15	q11.2	20820054-22681064	1861	NBEAP1, POTEB	arr[hg19] 15q11.2(20,820,054-22,681,064)x3
				22	q11.21	20109549-20259386	150	RANBP1, ZDHHC8, RTN4R	arr[hg19] 22q11.21(20,109,549-20,259,386)x3

P77/17	12.0	Bajau	male	22	q11.22	23111957-23258603	147	IGLL5, MIR650	arr[hg19] 22q11.22(23,111,957-23,258,603)x3
P84/17	10.0	Iban	male	14	q11.2	chr14:20010900-20423360	412	POTEM, LOC100508046, OR11H2, OR4Q3, OR4M1, OR4N2, OR4K3, OR4K2, OR4K5, OR4K1	arr[hg19] 14q11.2(20,010,900-20,423,360)x4
				15	q11.2	chr15:25416713-25497305	81	SNHG14	arr[hg19] 15q11.2(25,416,713-25,497,305)x4
				22	q11.22	chr22:22871747-23268151	396	PRAME, GGTLC2, MIR650, IGLL5	arr[hg19] 22q11.22(22,871,747-23,268,151)x3
				X	q21.31-q21.32	chrX:91691905-92043529	352	PCDH11X	arr[hg19] Xq21.31q21.32(91,691,905-92,043,529)x2
P87/17	5.0	Malay	male	14	q11.2	chr14:19562126-20423360	861	POTEG, POTEM,OR11H2, OR4Q3, OR4M1, OR4N2, OR4K3, OR4K2, OR4K5, OR4K1	arr[hg19] 14q11.2(19,562,126-20,423,360)x4
				15	q11.1-q11.2	chr15:20624193-22388224	1764	NBEAP1, POTEB	arr[hg19] 15q11.1q11.2(20,624,193-22,388,224)x3
				22	q11.22	chr22:22808987-23263262	454	ZNF280B, PRAME, GGTLC2, MIR650, IGLL5	arr[hg19] 22q11.22(22,808,987-23,263,262)x3
P110/17	6.6	Dusun	male	2	q11.2	chr2:97751996-98162177	410	ANKRD36, FAHD2B, ANKRD36B	arr[hg19] 2q11.2(97,751,996-98,162,177)x3
				5	q11.2	chr5:53317989-53813163	495	ARL15, SNX18, LINC01033	arr[hg19] 5q11.2(53,317,989-53,813,163)x3
				10	q26.3	chr10:135245766-135396272	151	SCART1, CYP2E1, SYCE1	arr[hg19] 10q26.3(135,245,766-135,396,272)x3
				15	q11.1-q11.2	chr15:20440525-22673387	2233	NBEAP1, POTEB	arr[hg19] 15q11.1q11.2(20,440,525-22,673,387)x3
				X	q21.31-q21.32	chrX:91691905-92115371	423	PCDH11X	arr[hg19] Xq21.31q21.32(91,691,905-92,115,371)x2
P250/17	2.0	Malay	male	22	q11.22	chr22:22871747-23258603	387	PRAME, GGTLC2, MIR650, IGLL5	arr[hg19] 22q11.22(22,871,747-23,258,603)x3
P258/17	15.0	Malay	female	14	q11.2	chr14:19790342-20413846	624	POTEM, OR11H2, OR4Q3, OR4M1, OR4N2, OR4K3, OR4K2, OR4K5, OR4K1	arr[hg19] 14q11.2(19,790,342-20,413,846)x3
				22	q11.22	chr22:23040137-23268562	228	MIR650, IGLL5	arr[hg19] 22q11.22(23,040,137-23,268,562)x3
P273/17	9.0	Chinese	male	14	q32.33	chr14:106224341-106927570	703	FAM30A	arr[hg19] 14q32.33(106,224,341-106,927,570)x3
				15	q11.2	chr15:25415167-25499916	85	SNHG14	arr[hg19] 15q11.2(25,415,167-25,499,916)x4
				22	q11.22	chr22:22871747-23268151	396	ZNF280B, PRAME, GGTLC2, MIR650, IGLL5	arr[hg19] 22q11.22(22,871,747-23,268,151)x3

				X	q21.31-q21.32	chrX:91691905-92358869	667	PCDH11X	arr[hg19] Xq21.31q21.32(91,691,905-92,358,869)x2
P310/17	1.7	Murut	female	2	q13	chr2:110826241-111084885	259	MALL, NPHP1, MTLN, LOC100507334	arr[hg19] 2q13(110,826,241-111,084,885)x4
				8	p23.1	chr8:7267352-7777159	510	DEFB103B, SPAG11B, FAM90A7P, FAM90A10P, DEFB4A	arr[hg19] 8p23.1(7,267,352-7,777,159)x3
				15	q11.2	chr15:20951442-22673387	1722	NBEAP1, POTEB	arr[hg19] 15q11.2(20,951,442-22,673,387)x3
				22	q11.22	chr22:22871747-23268562	397	PRAME, GGTLC2, MIR650, IGLL5	arr[hg19] 22q11.22(22,871,747-23,268,562)x3
P319/17	4.0	Malay	female	14	q11.1-q11.2	chr14:19002111-20423360	1421	POTEG, POTEM,OR11H2, OR4Q3, OR4M1, OR4N2, OR4K3, OR4K2, OR4K5, OR4K1	arr[hg19] 14q11.1q11.2(19,002,111-20,423,360)x3
				15	q11.1-q11.2	chr15:20646562-22320439	1674	NBEAP1, POTEB	arr[hg19] 15q11.1q11.2(20,646,562-22,320,439)x3
				22	q11.22	chr22:22895812-23268151	372	PRAME, GGTLC2, MIR650, IGLL5	arr[hg19] 22q11.22(22,895,812-23,268,151)x3
P321/17	15.0	Malay	male	15	q11.2	chr15:25415167-25502419	87	SNHG14	arr[hg19] 15q11.2(25,415,167-25,502,419)x4

Subject ID	Age at diagnosis	Ethnicity	Gender	Loss / Deletion					
				**Chr.**	**Cytoband**	**Location**	**Size (kb)**	**Gene(s)**	**Nomenclature**
P31/16	8.0	Malay	male	7	p14.1	38317557-38392024	74	TRG-AS1	arr[hg19] 7p14.1(38317557-38392024)x1
				8	p11.22	39235590-39386953	151	ADAM5, ADAM3A	arr[hg19] 8p11.22(39235590-39386953)x1
				16	p13.11	14980964-15116245	135	NOMO1, MIR3179-1, MIR3179-2, MIR3179-4, MIR3179-3, MIR3670-2, MIR3670-1, MIR3670-4, MIR3670-3, MIR3180-3, MIR3180-1, Q18MIR3180-2, PKD1P3-NPIPA1, LOC100288162, MIR6511A2, MIR6511A3, MIR6511A4, MIR6511A1, MIR6770-1, MIR6770-2, MIR6770-3, NPIPA1, PDXDC1	arr[hg19] 16p13.11(14,980,964-15,116,245)x1
				20	p12.1	14945736-15135938	190	MACROD2	arr[hg19] 20p12.1(14,945,736-15,135,938)x1
P478/16	5.0	Malay	female	2	q37.3	237224527-237387870	53	IQCA1	arr[hg19] 2q37.3(237,224,527-237,387,870)x1

				4	q13.2	69375335-69489323	111	UGT2B17	arr[hg19] 4q13.2(69,375,335-69,489,323)x1
				6	q14.1	78969052-79035173	66	n/a	arr[hg19] 6q14.1(78,969,052-79,035,173)x1
				16	p12.2	22631352-22730342	99	MIR548D2, MIR548AA2	arr[hg19] 16p12.2(22,631,352-22,730,342)x1
				17	p11.22	16621805-16722789	101	CCDC144A, USP32P1, FAM106CP	arr[hg19] 17p11.22(16,621,805-16,722,789)x1
P518/16	7.0	Chinese	male	4	q13.2	69375335-69489323	114	UGT2B17	arr[hg19] 4q13.2(69,375,335-69,489,323)x1
				9	p22.1	19446238-19565588	119	ACER2, SLC24A2	arr[hg19] 9p22.1(19,446,238-19,565,588)x1
P679/16	1.9	Malay	female	15	q21.3	53815592-55299086	1483	WDR72, UNC13C	arr[hg19] 15q21.3(53,815,592-55,299,086)x1
				15	q26.2	94923653-95574767	651	MCTP2	arr[hg19] 15q26.2(94,923,653-95,574,767)x1
P148/16	10.6	Chinese	male	4	q13.2	69375335-69485967	111	UGT2B17	arr[hg19] 4q13.2(69,375,335-69,485,967)x1
				7	p14.1	38290713-38386800	96	TARP, TRG-AS1	arr[hg19] 7p14.1(38,290,713-38,386,800)x1
P309/16	12.0	Sino Dusun	female	4	q13.2	69375335-69485967	111	UGT2B17	arr[hg19] 4q13.2(69,375,335-69,485,967)x1
				15	q11.2	22311797-22588019	276	LINC02203, LOC101927079, LOC101927079, OR4M2, OR4N4, OR4N3P, IGHV1OR15-1, LOC102724760, IGHV1OR15-3, LOC642131, MIR1268A, REREP3	arr[hg19] 15q11.2(22,311,797-22,588,019)x1
				15	q14	34723419-34810076	87	GOLGA8A	arr[hg19] 15q14(34,723,419-34,810,076)x1
				16	p13.11	14980964-15094696	114	NOMO1, MIR3179-4, MIR3179-1, MIR3179-3, MIR3179-2, MIR3670-1, MIR3670-4, MIR3670-2, MIR3670-3, MIR3180-1, MIR3180-3, MIR3180-2, PKD1P3-NPIPA1, LOC100288162, MIR6511A4, MIR6511A1, MIR6511A2, MIR6511A3, MIR6770-2, MIR6770-1, MIR6770-3, NPIPA1, PDXDC1	arr[hg19] 16p13.11(14,980,964-15,094,696)x1
P334/16	12.0	Malay	female	16	p13.11	15048755-15116245	67	PDXDC1	arr[hg19] 16p13.11(15,048,755-15,116,245)x1
				X	p11.23	48001282-48101968	101	SSX5	arr[hg19] Xp11.23(48,001,282-48,101,968)x1
P623/16	4.6	Brunei	female	1	q21.2	149036511-149123879	87	NBPF25P	arr[hg19] 1q21.2(149,036,511-149,123,879)x1
				7	q21.11	83124980-83181644	57	SEMA3E	arr[hg19] 7q21.11(83,124,980-83,181,644)x1

P577/16	10.0	Malay	male	7	q34	142331339-142493638	162	PRSS1	arr[hg19] 7q34(142,331,339-142,493,638)x1
P358/16	5.0	Chinese	female	4	q13.2	69375335-69489323	114	UGT2B17	arr[hg19] 4q13.2(69,375,335-69,489,323)x1
				4	q31.23	149369218-149917783	549	LOC105377480	arr[hg19] 4q31.23(149,369,218-149,917,783)x1
				6	p25.3	257340-379003	122	DUSP22	arr[hg19] 6p25.3(257,340-379,003)x1
				6	p21.33	31360094-31460220	100	MICA, HCP5, MICB	arr[hg19] 6p21.33(31,360,094-31,460,220)x1
				7	p14.1	38294088-38386800	93	TARP, TRG-AS1	arr[hg19] 7p14.1(38,294,088-38,386,800)x0
				7	q34	142350517-142493638	143	MTRNR2L6, PRSS1, PRSS3P2	arr[hg19] 7q34(142,350,517-142,493,638)x0
				9	p23	11734432-12069615	335	No gene	arr[hg19] 9p23(11,734,432-12,069,615)x1
				10	q21.3	68372084-68454889	83	CTNNA3	arr[hg19] 10q21.3(68,372,084-68,454,889)x1
				14	q11.2	22521884-23004788	483	LOC105370401	arr[hg19] 14q11.2(22,521,884-23,004,788)x1
				14	q32.33	106401663-107198332	797	FAM30A, ADAM6, LINC00226, LINC00221	arr[hg19] 14q32.33(106,401,663-107,198,332)x1
				22	q11.22	22381524-22599537	218	PRAMENP	arr[hg19] 22q11.22(22,381,524-22,599,537)x1
P636/16	8.0	Bajau	male	4	q13.2	69375335-69485967	111	UGT2B17	arr[hg19] 4q13.2(69,375,335-69,485,967)x1
				15	q11.1-q11.2	20395516-20986686	591	CHEK2P2, HERC2P3, GOLGA6L6, GOLGA8CP, NBEAP1	arr[hg19] 15q11.1q11.2(20,395,516-20,986,686)x1
				19	p12	20598430-20716377	118	ZNF826P	arr[hg19] 19p12(20,598,430-20,716,377)x1
				19	q13.2-q13.31	43294377-43542819	248	PSG10P, PSG1, PSG6, PSG7, PSG11	arr[hg19] 19q13.2q13.31(43,294,377-43,542,819)x1
P676/16	14.0	Malay	female	8	p11.22	39235590-39385182	150	ADAM5, ADAM3A	arr[hg19] 8p11.22(39,235,590-39,385,182)x1
				16	p13.11	14971402-15116245	145	NOMO1, NPIPA1, PDXDC1	arr[hg19] 16p13.11(14,971,402-15,116,245)x1
P772/16	4.0	Malay	male	4	q13.2	69375335-69484097	109	UGT2B17	arr[hg19] 4q13.2(69,375,335-69,484,097)x1
				10	q11.22	47546322-47652803	106	ANTXRLP1	arr[hg19] 10q11.22(47,546,322-47,652,803)x1
				14	q11.2	22862875-22955716	93	LOC105370401	arr[hg19] 14q11.2(22,862,875-22,955,716)x0

				22	q11.22	22381594-22551935	170	PRAMENP	arr[hg19] 22q11.22(22,381,594-22,551,935)x1
P521/16	14.0	Malay	male	4	q13.2	69375335-69505103	130	UGT2B17	arr[hg19] 4q13.2(69,375,335-69,505,103)x1
P32/16	1.8	Chinese	male	4	q13.2	69375335-69485967	111	UGT2B17	arr[hg19] 4q13.2(69,375,335-69,485,967)x1
				16	p12.2	21534303-21839340	305	METTL9, OTOA	arr[hg19] 16p12.2(21,534,303-21,839,340)x1
P35/16	4.0	Chinese	male	16	p13.11	14989850-15116245	63	NPIPA1, PDXDC1	arr[hg19] 16p13.11(14,989,850-15,116,245)x1
P80/16	1.9	Chinese	male	9	p21.1	28527185-28631097	104	LINGO2	arr[hg19] 9p21.1(28,527,185-28,631,097)x1
P162/16	5.6	Malay	male	3	p14.2	60084923-60666906	582	FHIT	arr[hg19] 3p14.2(60,084,923-60,666,906)x1
				7	q34	142331339-142493638	162	PRSS1	arr[hg19] 7q34(142,331,339-142,493,638)x1
				16	p13.11	14980964-15116245	135	NPIPA1, PDXDC1	arr[hg19] 16p13.11(14,980,964-15,116,245)x1
				18	p11.32	273215-557564	284	COLEC12	arr[hg19] 18p11.32(273,215-557,564)x1
P311/16	3.6	Kadazan	female	4	q13.2	69375335-69485967	111	UGT2B17	arr[hg19] 4q13.2(69,375,335-69,485,967)x1
P350/16	9.0 m	Dusun	male	19	q13.31	43458041-43784309	326	PSG11, PSG2, PSG5, PSG4, PSG9	arr[hg19] 19q13.31(43,458,041-43,784,309)x1
P314/16	14.0	Malay	female	4	q13.2	69375335-69489323	114	UGT2B17	arr[hg19] 4q13.2(69,375,335-69,489,323)x1
P281/16	14.0	Melanau	male	8	q24.21	129211399-130032394	821	LINC00824	arr[hg19] 8q24.21(129,211,399-130,032,394)x0
				18	q21.32	57568944-57728033	159	PMAIP1	arr[hg19] 18q21.32(57,568,944-57,728,033)x1
P67/16	3.8	Malay	male	7	q34	142118033-142474939	357	MTRNR2L6, PRSS1	arr[hg19] 7q34(142,118,033-142,474,939)x1
				8	p23.1	7222168-7776618	554	DEFB4B, DEFB103A, DEFB103B, SPAG11B, DEFB104B, DEFB104A, DEFB106A, DEFB106B, DEFB105A, DEFB105B, DEFB107A, DEFB107B, PRR23D1, PRR23D2, FAM90A7P, FAM90A10P, PRR23D2, PRR23D1, DEFB107A, DEFB107B, DEFB105B, DEFB105A, DEFB106A, DEFB106B, DEFB104A, DEFB104B, SPAG11B, SPAG11A, DEFB103A, DEFB103B, DEFB4A	arr[hg19] 8p23.1(7,222,168-7,776,618)x1
				14	q11.2	22894252-22997833	104	LOC105370401	arr[hg19] 14q11.2(22,894,252-22,997,833)x1

				14	q32.33	106401458-107179847	242	FAM30A, ADAM6, LINC00226, LINC00221	arr[hg19] 14q32.33(106,401,458-107,179,847)x1
P359/16	4.0	Malay	male	2	q32.1	185699922-186614062	914	ZNF804A, FSIP2	arr[hg19] 2q32.1(185,699,922-186,614,062)x1
				3	q26.32	176906008-177196371	290	TBL1XR1, LINC00578, LINC00501	arr[hg19] 3q26.32(176,906,008-177,196,371)x1
				4	q13.2	69375335-69489323	114	UGT2B17	arr[hg19] 4q13.2(69,375,335-69,489,323)x1
				7	p14.1	38332293-38398047	66	TRG-AS1	arr[hg19] 7p14.1(38,332,293-38,398,047)x1
				7	q34	142337299-142493638	156	PRSS1	arr[hg19] 7q34(142,337,299-142,493,638)x1
				12	q15	69284267-69834189	550	CPM, CPSF6, YEATS4, LYZ	arr[hg19] 12q15(69,284,267-69,834,189)x1
				12	q21.1	74316836-75108308	791	LOC100507377, ATXN7L3B	arr[hg19] 12q21.1(74,316,836-75,108,308)x1
				16	p13.11	15049968-15116245	66	PDXDC1	arr[hg19] 16p13.11(15,049,968-15,116,245)x1
				18	q12.1	26243375-27472920	1230	Intron	arr[hg19] 18q12.1(26,243,375-27,472,920)x1
				19	q13.2-q13.31	43294377-43519362	225	PSG10P, PSG1, PSG6, PSG7, PSG11	arr[hg19] 19q13.2q13.31(43,294,377-43,519,362)x1
P461/06	2.9	Malay	male	1	q31.1	189324091-189537319	213	Intron	arr[hg19] 1q31.1(189,324,091-189,537,319)x1
				4	q13.2	69375335-69484097	109	UGT2B17	arr[hg19] 4q13.2(69,375,335-69,484,097)x1
				4	q34.3	179616621-179714047	97	Intron	arr[hg19] 4q34.3(179,616,621-179,714,047)x1
P474/16	6.0	Malay	male	15	q11.1-q11.2	20541967-22588019	2046	NBEAP1, POTEB	arr[hg19] 15q11.1q11.2(20,541,967-22,588,019)x1
P481/16	1.0	Bugis	male	7	q21.3	93928259-94033337	105	COL1A2, COL1A2-AS1	arr[hg19] 7q21.3(93,928,259-94,033,337)x1
				7	q21.3	94076173-94279344	203	CASD1, SGCE	arr[hg19] 7q21.3(94,076,173-94,279,344)x1
				14	q11.2	22543577-22917633	374	LOC105370401	arr[hg19] 14q11.2(22,543,577-22,917,633)x1
				14	q32.33	106801749-107205727	404	LINC00221	arr[hg19] 14q32.33(106,801,749-107,205,727)x1
				15	q11.1-q11.2	20291300-21142256	851	NBEAP1	arr[hg19] 15q11.1q11.2(20,291,300-21,142,256)x1

				15	q11.2	21909390-22681064	772	POTEB	arr[hg19] 15q11.2(21,909,390-22,681,064)x1
				20	q11.22	33392969-33566329	173	NCOA6, GGT7, ACSS2, GSS, MYH7B	arr[hg19] 20q11.22(33,392,969-33,566,329)x1
				22	q11.22	22557032-22627044	70	VPREB1	arr[hg19] 22q11.22(22,557,032-22,627,044)x1
P661/16	9.0	Malay	male	4	q13.2	69375335-69505103	130	UGT2B17	arr[hg19] 4q13.2(69,375,335-69,505,103)x1
				7	p14.1	38310710-38391353	81	TARP	arr[hg19] 7p14.1(38,310,710-38,391,353)x1
				7	q34	142331339-142493638	162	PRSS1	arr[hg19] 7q34(142,331,339-142,493,638)x1
				14	q11.2	22892460-23003305	111	LOC105370401	arr[hg19] 14q11.2(22,892,460-23,003,305)x1
				14	q32.33	106460889-107217878	757	LINC00221, LINC00226	arr[hg19] 14q32.33(106,460,889-107,217,878)x1
				15	q14	34700670-34813107	112	GOLGA8A	arr[hg19] 15q14(34,700,670-34,813,107)x1
				17	q11.2	28634241-28785922	152	TMIGD1, CPD	arr[hg19] 17q11.2(28,634,241-28,785,922)x1
P809/16	6.0	Bidayuh	male	1	q21.2	149036511-149436830	400	FCGR1CP	arr[hg19] 1q21.2(149,036,511-149,436,830)x1
				1	q31.2	190807349-191466271	659	LINC01680	arr[hg19] 1q31.2(190,807,349-191,466,271)x1
				1	q31.3	194580717-194691336	111	Intron	arr[hg19] 1q31.3(194,580,717-194,691,336)x1
				3	q26.1	162513446-162623885	110	Intron	arr[hg19] 3q26.1(162,513,446-162,623,885)x1
				3	q26.32	176914480-177197754	283	LINC00501, LINC00578	arr[hg19] 3q26.32(176,914,480-177,197,754)x1
				4	q13.2	69375335-69489323	114	UGT2B17	arr[hg19] 4q13.2(69,375,335-69,489,323)x1
				7	p14.1	38311097-38371334	60	TARP	arr[hg19] 7p14.1(38,311,097-38,371,334)x0
				12	p13.1	14443118-14564645	122	ATF7IP	arr[hg19] 12p13.1(14,443,118-14,564,645)x1
				13	q14.11	44725467-45007697	282	SERP2, TSC22D1	arr[hg19] 13q14.11(44,725,467-45,007,697)x1
				14	q11.2	22892460-22999617	107	LOC105370401	arr[hg19] 14q11.2(22,892,460-22,999,617)x1
				14	q32.33	106401663-107200888	799	Intron	arr[hg19] 14q32.33(106,401,663-107,200,888)x1
				22	q11.22	22382095-22520067	138		arr[hg19] 22q11.22(22,382,095-22,520,067)x1

				X	p11.4	41781813-42124810	343	CASK	arr[hg19] Xp11.4(41,781,813-42,124,810)x0
P669/16	1.4	Malay	female	4	q13.2	69375335-69485967	111	UGT2B17	arr[hg19] 4q13.2(69,375,335-69,485,967)x1
				7	p14.1	38290713-38352227	62	TARP	arr[hg19] 7p14.1(38,290,713-38,352,227)x1
P774/16	16.0	Chinese	female	4	q13.2	69375335-69485967	111	UGT2B17	arr[hg19] 4q13.2(69,375,335-69,485,967)x1
				7	p14.1	38290713-38352227	62	TARP	arr[hg19] 7p14.1(38,290,713-38,352,227)x0
				7	q34	142338976-142493638	155	PRSS1	arr[hg19] 7q34(142,338,976-142,493,638)x1
				13	q12.3	30999207-31131615	132	UBE2L5, HMGB1	arr[hg19] 13q12.3(30,999,207-31,131,615)x1
				13	q14.11	44725467-45011829	286	SMIM2, SERP2, TSC22D1	arr[hg19] 13q14.11(44,725,467-45,011,829)x1
				14	q32.33	106923068-107200888	278	LINC00221	arr[hg19] 14q32.33(106,923,068-107,200,888)x1
				22	q11.22	22457086-22520067	63	Intron	arr[hg19] 22q11.22(22,457,086-22,520,067)x1
P503/16	2.0	Malay	male	4	q13.2	69375335-69489323	114	UGT2B17	arr[hg19] 4q13.2(69,375,335-69,489,323)x1
				7	q21.3	92995751-93150201	154	CALCR	arr[hg19] 7q21.3(92,995,751-93,150,201)x1
				16	p13.11	15049872-15116245	66	PDXDC1	arr[hg19] 16p13.11(15,049,872-15,116,245)x1
P690/16	3.5	Rungus	male	4	q13.2	69375335-69489323	114	UGT2B17	arr[hg19] 4q13.2(69,375,335-69,489,323)x1
				7	q35	143223905-143568047	344	TCAF2, TCAF1, CTAGE15, CTAGE6	arr[hg19] 7q35(143,223,905-143,568,047)x1
P687/16	6.0	Malay	female	4	q13.2	69375335-69489323	114	UGT2B17	arr[hg19] 4q13.2(69,375,335-69,489,323)x1
P5/17	2.1	Sungai	male	11	q25	134355896-134472198	116	LOC283177	arr[hg19] 11q25(134,355,896-134,472,198)x1
				15	q14	34722789-34810076	87	GOLGA8A	arr[hg19] 15q14(34,722,789-34,810,076)x1
				19	p12	20596193-20716377	120	Intron	arr[hg19] 19p12(20,596,193-20,716,377)x0
P7/17	4.6	Indian	female	3	p23	31402007-31700236	298	STT3B	arr[hg19] 3p23(31,402,007-31,700,236)x1
				8	p21.3	21160599-21585379	425	GFRA2	arr[hg19] 8p21.3(21,160,599-21,585,379)x1
				14	q11.2	22883938-22978775	95	LOC105370401	arr[hg19] 14q11.2(22,883,938-22,978,775)x0

				16	p13.11	15048755-15116245	67	PDXDC1	arr[hg19] 16p13.11(15,048,755-15,116,245)x1
				18	q12.3	38451291-38590011	139	Intron	arr[hg19] 18q12.3(38,451,291-38,590,011)x1
P8/17	6.6	Iban	female	4	q13.2	69374928-69485967	111	UGT2B17	arr[hg19] 4q13.2(69,374,928-69,485,967)x1
				7	q34	142331339-142486033	155	PRSS1	arr[hg19] 7q34(142,331,339-142,486,033)x1
				13	q21.32	67255536-67722028	466	PCDH9	arr[hg19] 13q21.32(67,255,536-67,722,028)x1
P9/17	4.8	Iban	male	4	q13.2	69375335-69485967	111	UGT2B17	arr[hg19] 4q13.2(69,375,335-69,485,967)x1
				6	p21.2	37038431-37154223	116	PIM1	arr[hg19] 6p21.2(37,038,431-37,154,223)x1
				7	p14.1	38294088-38387431	93	TARP, TRG-AS1	arr[hg19] 7p14.1(38,294,088-38,387,431)x1
				7	q34	142351749-142493638	142	PRSS1	arr[hg19] 7q34(142,351,749-142,493,638)x1
				14	q11.2	22367537-23005786	638	LOC105370401	arr[hg19] 14q11.2(22,367,537-23,005,786)x1
				14	q32.33	106401663-107179847	778	LINC00221, LINC00226	arr[hg19] 14q32.33(106,401,663-107,179,847)x1
				16	p13.11	15048755-15125441	77	PDXDC1	arr[hg19] 16p13.11(15,048,755-15,125,441)x1
				18	q21.2	53256580-53526945	270	TCF4, LINC01415	arr[hg19] 18q21.2(53,256,580-53,526,945)x1
				22	q11.22	22517825-22599537	82	VPREB1	arr[hg19] 22q11.22(22,517,825-22,599,537)x1
P55/17	11.0	Chinese	male	14	q32.33	106401458-106679068	278	Intron	arr[hg19] 14q32.33(106,401,458-106,679,068)x1
P77/17	12.0	Bajau	male	4	q13.2	69375335-69485967	111	UGT2B17	arr[hg19] 4q13.2(69,375,335-69,485,967)x1
				11	q11	55374019-55452997	79	OR4P4, OR4S2, OR4C6	arr[hg19] 11q11(55,374,019-55,452,997)x0
				16	p13.11	15048755-15116245	67	PDXDC1	arr[hg19] 16p13.11(15,048,755-15,116,245)x1
				16	p11.2	32380938-33338112	957	TP53TG3	arr[hg19] 16p11.2(32,380,938-33,338,112)x1
P84/17	10.0	Iban	male	3	q13.2	chr3:112055011-112220798	166	CD200, BTLA	arr[hg19] 3q13.2(112,055,011-112,220,798)x1
				3	q25.2	chr3:152905654-153876379	971	LINC02006, ARHGEF26-AS1, ARHGEF26	arr[hg19] 3q25.2(152,905,654-153,876,379)x1
				3	q26.1	chr3:165807559-166743282	936	Intron	arr[hg19] 3q26.1(165,807,559-166,743,282)x1

				3	q26.3	chr3:173332639-173547620	215	NLGN1	arr[hg19] 3q26.31(173,332,639-173,547,620)x1
				6	q16.3	chr6:102749478-103137441	388	Intron	arr[hg19] 6q16.3(102,749,478-103,137,441)x1
				7	p14.1	chr7:38294088-38398047	104	TARP, TRG-AS1	arr[hg19] 7p14.1(38,294,088-38,398,047)x0
				7	q34	chr7:142331339-142493638	162	PRSS1	arr[hg19] 7q34(142,331,339-142,493,638)x1
				9	p24.1	chr9:7470752-7860465	390	DMAC1	arr[hg19] 9p24.1(7,470,752-7,860,465)x1
				9	p21.1	chr9:28774630-29206194	432	LINGO2, MIR873	arr[hg19] 9p21.1(28,774,630-29,206,194)x1
				13	q12.12	chr13:23395158-23666489	271	LINC00621	arr[hg19] 13q12.12(23,395,158-23,666,489)x1
				14	q11.2	chr14:22862875-22975820	113	LOC105370401	arr[hg19] 14q11.2(22,862,875-22,975,820)x1
				15	q14	chr15:34722789-34810076	87	GOLGA8A	arr[hg19] 15q14(34,722,789-34,810,076)x1
				19	q13.31	chr19:43454617-43786678	332	PSG11, PSG2, PSG5, PSG4, PSG9	arr[hg19] 19q13.31(43,454,617-43,786,678)x1
				20	q12	chr20:39379194-39579664	200	Intron	arr[hg19] 20q12(39,379,194-39,579,664)x1
P87/17	5.0	Malay	male	3	q26.1	chr3:162513446-162623885	110	Intron	arr[hg19] 3q26.1(162,513,446-162,623,885)x1
				4	q13.2	chr4:69375335-69485967	111	UGT2B17	arr[hg19] 4q13.2(69,375,335-69,485,967)x1
				6	p25.3	chr6:257340-379003	122	DUSP22	arr[hg19] 6p25.3(257,340-379,003)x1
				7	p14.1	chr7:38286192-38358956	73	TARP	arr[hg19] 7p14.1(38,286,192-38,358,956)x1
				11	p14.3	chr11:21863340-22007028	144	Intron	arr[hg19] 11p14.3(21,863,340-22,007,028)x1
				14	q11.2	chr14:22829384-22997524	168	LOC105370401	arr[hg19] 14q11.2(22,829,384-22,997,524)x1
				15	q14	chr15:34727609-34814530	87	GOLGA8A	arr[hg19] 15q14(34,727,609-34,814,530)x1
P110/17	6.6	Dusun	male	1	q31.2	chr1:190815815-190907710	92	Intron	arr[hg19] 1q31.2(190,815,815-190,907,710)x0
				2	p11.2	chr2:89130740-89618595	488	Intron	arr[hg19] 2p11.2(89,130,740-89,618,595)x1
				3	q13.2	chr3:112044034-112211801	168	CD200, BTLA	arr[hg19] 3q13.2(112,044,034-112,211,801)x1
				3	q26.32	chr3:177081506-177554204	473	Intron	arr[hg19] 3q26.32(177,081,506-177,554,204)x1

				5	p15.2	chr5:10962721-11173649	211	CTNND2	arr[hg19] 5p15.2(10,962,721-11,173,649)x1
				7	p14.1	chr7:38311097-38398047	87	TARP	arr[hg19] 7p14.1(38,311,097-38,398,047)x0
				7	q34	chr7:142331339-142493638	162	PRSS1	arr[hg19] 7q34(142,331,339-142,493,638)x0
				8	p23.1	chr8:7297327-7747687	450	DEFB103B, SPAG11B, FAM90A7P, FAM90A10P, DEFB4A, DEFB105B	arr[hg19] 8p23.1(7,297,327-7,747,687)x1
				14	q11.2	chr14:22617414-23000062	383	LOC105370401	arr[hg19] 14q11.2(22,617,414-23,000,062)x1
				14	q32.33	chr14:106401663-107200888	799	LINC00221, LINC00226	arr[hg19] 14q32.33(106,401,663-107,200,888)x0
				15	q11.2	chr15:24516860-24706906	190	PWRN3	arr[hg19] 15q11.2(24,516,860-24,706,906)x1
				15	q14	chr15:34722631-34810076	87	GOLGA8A	arr[hg19] 15q14(34,722,631-34,810,076)x1
P250/17	2.0	Malay	male	12	q21.31	chr12:85046713-85437197	390	SLC6A15	arr[hg19] 12q21.31(85,046,713-85,437,197)x1
				14	q32.33	chr14:106401458-106802041	401	Intron	arr[hg19] 14q32.33(106,401,458-106,802,041)x1
				15	q14	chr15:34700670-34800595	100	GOLGA8A	arr[hg19] 15q14(34,700,670-34,800,595)x1
				22	q11.22	chr22:22517825-22601814	84	VPREB1	arr[hg19] 22q11.22(22,517,825-22,601,814)x1
P258/17	15.0	Malay	female	4	q13.2	chr4:70127631-70232112	104	UGT2B28	arr[hg19] 4q13.2(70,127,631-70,232,112)x0
				9	q34.11	chr9:133346503-133461084	115	ASS1, FUBP3	arr[hg19] 9q34.11(133,346,503-133,461,084)x1
				13	q34	chr13:114847012-115108385	261	RASA3, CDC16, UPF3A, CHAMP1	arr[hg19] 13q34(114,847,012-115,108,385)x1
				15	q14	chr15:34700670-34814530	114	GOLGA8A	arr[hg19] 15q14(34,700,670-34,814,530)x1
				16	p13.11	chr16:15048755-15116245	67	PDXDC1	arr[hg19] 16p13.11(15,048,755-15,116,245)x1
P273/17	9.0	Chinese	male	4	q13.2	chr4:69375335-69489323	114	UGT2B17	arr[hg19] 4q13.2(69,375,335-69,489,323)x1
P310/17	1.7	Murut	female	4	q13.2	chr4:69375335-69489323	114	UGT2B17	arr[hg19] 4q13.2(69,375,335-69,489,323)x1
				10	q11.22	chr10:47058779-47392320	334	NPY4R, LINC00842, ANXA8, AGAP9	arr[hg19] 10q11.22(47,058,779-47,392,320)x1
				15	q14	chr15:34695309-34810076	115	GOLGA8A	arr[hg19] 15q14(34,695,309-34,810,076)x1
P319/17	4.0	Malay	female	4	q13.2	chr4:69375335-69489323	114	UGT2B17	arr[hg19] 4q13.2(69,375,335-69,489,323)x1

				5	p13.2	chr5:36739295-37021956	283	NIPBL	arr[hg19] 5p13.2(36,739,295-37,021,956)x1
				7	q34	chr7:142410454-142493638	83	PRSS1	arr[hg19] 7q34(142,410,454-142,493,638)x1
				13	q12.11	chr13:19543241-19622143	79	LINC00442	arr[hg19] 13q12.11(19,543,241-19,622,143)x1
				14	q11.2	chr14:22913993-22988571	75	LOC105370401	arr[hg19] 14q11.2(22,913,993-22,988,571)x0
				14	q32.33	chr14:106401663-107171764	770	FAM30A, ADAM6, LINC00226, LINC00221	arr[hg19] 14q32.33(106,401,663-107,171,764)x1
				16	p13.11	chr16:14939327-15087165	148	NOMO1, NPIPA1, PDXDC1	arr[hg19] 16p13.11(14,939,327-15,087,165)x1
				22	q11.22	chr22:22382095-22520067	138	Intron	arr[hg19] 22q11.22(22,382,095-22,520,067)x1
P321/17	15.0	Malay	male	3	q26.1	chr3:162512644-162623885	111	Intron	arr[hg19] 3q26.1(162,512,644-162,623,885)x1
				4	q13.2	chr4:69375335-69485967	111	UGT2B17	arr[hg19] 4q13.2(69,375,335-69,485,967)x1
				6	p25.3	chr6:257340-379003	122	DUSP22	arr[hg19] 6p25.3(257,340-379,003)x1
				7	q34	chr7:142274853-142493638	219	PRSS1	arr[hg19] 7q34(142,274,853-142,493,638)x0
				15	q13.2	chr15:30493348-31082263	589	CHRFAM7A, ARHGAP11B	arr[hg19] 15q13.2(30,493,348-31,082,263)x1
				15	q13.31	chr15:32444195-32876972	433	CHRNA7, GOLGA8K, GOLGA8O	arr[hg19] 15q13.3(32,444,195-32,876,972)x1
				16	p13.11	chr16:14978956-15116245	137	NOMO1, NPIPA1, PDXDC1	arr[hg19] 16p13.11(14,978,956-15,116,245)x1
