# Supplementary material for: Single Nucleotide Polymorphism Microarray Analysis Unveils Copy‐Number Abnormalities and Genetic Heterogeneity in Malaysian Childhood B‐Cell Precursor Acute Lymphoblastic Leukemia
Source: Mol Genet Genomic Med. 2026 Mar 2;14(3):e70182. doi: 10.1002/mgg3.70182 (PMC12953716; doi:10.1002/mgg3.70182)
Supplement: Supplementary file 4 — Table S3: SNP 6.0 microarray and MLPA P327 results of chromosome 21 in 55 Malaysian patients with BCP‐ALL. The results showed no chromothripsis occurred in any patients. [file MGG3-14-e70182-s003.docx]

**Supporting Information Table 3**: SNP 6.0 microarray and MLPA P327 results of chromosome 21 in 55 Malaysian patients with BCP-ALL. The results showed no chromothripsis occurred in any patients.

| **Subject ID** | **SNP 6.0 microarray** | **MLPA P327** | | | | | | | | | | | | | | | | | | | | | | | | | | | | |
| --- | --- | --- | --- | --- | --- | --- | --- | --- | --- | --- | --- | --- | --- | --- | --- | --- | --- | --- | --- | --- | --- | --- | --- | --- | --- | --- | --- | --- | --- | --- |
|  |  | **HSPA13 (21q11.2)** | **SAMSN1 (21q11.2)** | **MIR99A (21q21.1)** | **BTG3 (21q21.1)** | **TMPRSS15 (21q21.1)** | **NCAM2 (21q21.1)** | **MIR155 (21q21.1)** | **APP (21q21.3)** | **CYYR1 (21q21.3)** | **ADAMTS5 (21q21.3)** | **BACH1 (21q21.3)** | **TIAM1 (21q22.11)** | **OLIG2 (21q22.11)** | **KCNE2 (21q22.11)** | **RUNX1 (21q22.12)** | **SIM2 (21q22.13)** | **HLCS (21q22.13)** | **DYRK1A (21q22.13)** | **KCNJ6 (21q22.13)** | **ERG (21q22.2)** | **ETS2 (21q22.2)** | **PSMG1 (21q22.2)** | **TMPRSS2 (21q22.3)** | **RIPK4 (21q22.3)** | **TFF1 (21q22.3)** | **ITGB2 (21q22.3)** | **SLC19A1 (21q22.3)** | **COL6A2 (21q22.3)** | **PRMT2 (21q22.3)** |
| 1/16 | No detection | | | | | | | | | | | | | | | | | | | | | | | | | | | | | |
| 31/16 | Whole chromosome gain |  |  |  |  |  |  |  |  |  |  |  |  |  |  |  |  |  |  |  |  |  |  |  |  |  |  |  |  |  |
| 32/16 | Whole chromosome gain |  |  |  |  |  |  |  |  |  |  |  |  |  |  |  |  |  |  |  |  |  |  |  |  |  |  |  |  |  |
| 35/16 | No detection | | | | | | | | | | | | | | | | | | | | | | | | | | | | | |
| 67/16 | No detection | | | | | | | | | | | | | | | | | | | | | | | | | | | | | |
| 80/16 | No detection | | | | | | | | | | | | | | | | | | | | | | | | | | | | | |
| 148/16 | Whole chromosome gain |  |  |  |  |  |  |  |  |  |  |  |  |  |  |  |  |  |  |  |  |  |  |  |  |  |  |  |  |  |
| 162/16 | Whole chromosome gain |  |  |  |  |  |  |  |  |  |  |  |  |  |  |  |  |  |  |  |  |  |  |  |  |  |  |  |  |  |
| 242/16 | Whole chromosome gain |  |  |  |  |  |  |  |  |  |  |  |  |  |  |  |  |  |  |  |  |  |  |  |  |  |  |  |  |  |
| 281/16 | Intragenic ERG loss  (47 kb) |  |  |  |  |  |  |  |  |  |  |  |  |  |  |  |  |  |  |  |  |  |  |  |  |  |  |  |  |  |
| 300/16 | No detection | | | | | | | | | | | | | | | | | | | | | | | | | | | | | |
| 309/16 | No detection | | | | | | | | | | | | | | | | | | | | | | | | | | | | | |
| 310/16 | Whole chromosome gain |  |  |  |  |  |  |  |  |  |  |  |  |  |  |  |  |  |  |  |  |  |  |  |  |  |  |  |  |  |
| 314/16 | No detection | | | | | | | | | | | | | | | | | | | | | | | | | | | | | |
| 334/16 | No detection | | | | | | | | | | | | | | | | | | | | | | | | | | | | | |
| 350/16 | No detection | | | | | | | | | | | | | | | | | | | | | | | | | | | | | |
| 358/16 | No detection | | | | | | | | | | | | | | | | | | | | | | | | | | | | | |
| 359/16 | No detection | | | | | | | | | | | | | | | | | | | | | | | | | | | | | |
| 429/16 | No detection | | | | | | | | | | | | | | | | | | | | | | | | | | | | | |
| 461/16 | No detection | | | | | | | | | | | | | | | | | | | | | | | | | | | | | |
| 474/16 | Whole chromosome gain |  |  |  |  |  |  |  |  |  |  |  |  |  |  |  |  |  |  |  |  |  |  |  |  |  |  |  |  |  |
| 478/16 | Whole chromosome gain |  |  |  |  |  |  |  |  |  |  |  |  |  |  |  |  |  |  |  |  |  |  |  |  |  |  |  |  |  |
| 481/16 | No detection | | | | | | | | | | | | | | | | | | | | | | | | | | | | | |
| 503/16 | Whole chromosome gain |  |  |  |  |  |  |  |  |  |  |  |  |  |  |  |  |  |  |  |  |  |  |  |  |  |  |  |  |  |
| 518/16 | Whole chromosome gain |  |  |  |  |  |  |  |  |  |  |  |  |  |  |  |  |  |  |  |  |  |  |  |  |  |  |  |  |  |
| 521/16 | No detection |  |  |  |  |  |  |  |  |  |  |  |  |  |  |  |  |  |  |  |  |  |  |  |  |  |  |  |  |  |
| 527/16 | Whole chromosome gain |  |  |  |  |  |  |  |  |  |  |  |  |  |  |  |  |  |  |  |  |  |  |  |  |  |  |  |  |  |
| 577/16 | Intragenic ERG loss  (42 kb) |  |  |  |  |  |  |  |  |  |  |  |  |  |  |  |  |  |  |  |  |  |  |  |  |  |  |  |  |  |
| 623/16 | No detection | | | | | | | | | | | | | | | | | | | | | | | | | | | | | |
| 636/16 | No detection | | | | | | | | | | | | | | | | | | | | | | | | | | | | | |
| 661/16 | No detection | | | | | | | | | | | | | | | | | | | | | | | | | | | | | |
| 669/16 | Whole chromosome gain |  |  |  |  |  |  |  |  |  |  |  |  |  |  |  |  |  |  |  |  |  |  |  |  |  |  |  |  |  |
| 676/16 | No detection | | | | | | | | | | | | | | | | | | | | | | | | | | | | | |
| 679/16 | Whole chromosome gain |  |  |  |  |  |  |  |  |  |  |  |  |  |  |  |  |  |  |  |  |  |  |  |  |  |  |  |  |  |
| 687/16 | Whole chromosome gain |  |  |  |  |  |  |  |  |  |  |  |  |  |  |  |  |  |  |  |  |  |  |  |  |  |  |  |  |  |
| 690/16 | Whole chromosome gain |  |  |  |  |  |  |  |  |  |  |  |  |  |  |  |  |  |  |  |  |  |  |  |  |  |  |  |  |  |
| 731/16 | Whole chromosome gain |  |  |  |  |  |  |  |  |  |  |  |  |  |  |  |  |  |  |  |  |  |  |  |  |  |  |  |  |  |
| 772/16 | No detection | | | | | | | | | | | | | | | | | | | | | | | | | | | | | |
| 774/16 | No detection | | | | | | | | | | | | | | | | | | | | | | | | | | | | | |
| 809/16 | No detection | | | | | | | | | | | | | | | | | | | | | | | | | | | | | |
| 5/17 | Whole chromosome gain |  |  |  |  |  |  |  |  |  |  |  |  |  |  |  |  |  |  |  |  |  |  |  |  |  |  |  |  |  |
| 7/17 | No detection | | | | | | | | | | | | | | | | | | | | | | | | | | | | | |
| 8/17 | No detection | | | | | | | | | | | | | | | | | | | | | | | | | | | | | |
| 9/17 | No detection | | | | | | | | | | | | | | | | | | | | | | | | | | | | | |
| 55/17 | No detection | | | | | | | | | | | | | | | | | | | | | | | | | | | | | |
| 77/17 | No detection | | | | | | | | | | | | | | | | | | | | | | | | | | | | | |
| 84/17 | Intragenic ERG loss  (52 kb) |  |  |  |  |  |  |  |  |  |  |  |  |  |  |  |  |  |  |  |  |  |  |  |  |  |  |  |  |  |
| 87/17 | Whole chromosome gain |  |  |  |  |  |  |  |  |  |  |  |  |  |  |  |  |  |  |  |  |  |  |  |  |  |  |  |  |  |
| 110/17 | No detection | | | | | | | | | | | | | | | | | | | | | | | | | | | | | |
| 250/17 | Whole chromosome gain |  |  |  |  |  |  |  |  |  |  |  |  |  |  |  |  |  |  |  |  |  |  |  |  |  |  |  |  |  |
| 258/17 | No detection | | | | | | | | | | | | | | | | | | | | | | | | | | | | | |
| 273/17 | No detection | | | | | | | | | | | | | | | | | | | | | | | | | | | | | |
| 310/17 | Whole chromosome gain |  |  |  |  |  |  |  |  |  |  |  |  |  |  |  |  |  |  |  |  |  |  |  |  |  |  |  |  |  |
| 319/17 | Whole chromosome gain |  |  |  |  |  |  |  |  |  |  |  |  |  |  |  |  |  |  |  |  |  |  |  |  |  |  |  |  |  |
| 321/17 | No detection | | | | | | | | | | | | | | | | | | | | | | | | | | | | | |

*Legend: Blue – Gain; Red – Loss.
